# Pregnancy and Infant Outcomes among HIV-Infected Women Taking Long-Term ART with and without Tenofovir in the DART Trial

**DOI:** 10.1371/journal.pmed.1001217

**Published:** 2012-05-15

**Authors:** Diana M. Gibb, Hilda Kizito, Elizabeth C. Russell, Ennie Chidziva, Eva Zalwango, Ruth Nalumenya, Moira Spyer, Dinah Tumukunde, Kusum Nathoo, Paula Munderi, Hope Kyomugisha, James Hakim, Heiner Grosskurth, Charles F. Gilks, A. Sarah Walker, Phillipa Musoke, on behalf of the DART trial team

**Affiliations:** 1 MRC Clinical Trials Unit, London, United Kingdom; 2 Joint Clinical Research Centre, Kampala, Uganda; 3 University of Zimbabwe Clinical Research Centre, College of Health Sciences, Harare, Zimbabwe; 4 MRC/UVRI Uganda Research Unit on AIDS, Entebbe, Uganda; 5 Infectious Diseases Institute, Makerere University, Mulago, Uganda; 6 University of Zimbabwe Medical School, Department of Paediatrics and Child Health, Harare, Zimbabwe; 7 Imperial College, London, United Kingdom; 8 Department of Paediatrics and Child Health, College of Health Sciences, Makerere University, Uganda; University of Cape Town, South Africa

## Abstract

**Background:**

Few data have described long-term outcomes for infants born to HIV-infected African women taking antiretroviral therapy (ART) in pregnancy. This is particularly true for World Health Organization (WHO)–recommended tenofovir-containing first-line regimens, which are increasingly used and known to cause renal and bone toxicities; concerns have been raised about potential toxicity in babies due to in utero tenofovir exposure.

**Methods and Findings:**

Pregnancy outcome and maternal/infant ART were collected in Ugandan/Zimbabwean HIV-infected women initiating ART during The Development of AntiRetroviral Therapy in Africa (DART) trial, which compared routine laboratory monitoring (CD4; toxicity) versus clinically driven monitoring. Women were followed 15 January 2003 to 28 September 2009. Infant feeding, clinical status, and biochemistry/haematology results were collected in a separate infant study. Effect of in utero ART exposure on infant growth was analysed using random effects models.

382 pregnancies occurred in 302/1,867 (16%) women (4.4/100 woman-years [95% CI 4.0–4.9]). 226/390 (58%) outcomes were live-births, 27 (7%) stillbirths (≥22 wk), and 137 (35%) terminations/miscarriages (<22 wk). Of 226 live-births, seven (3%) infants died <2 wk from perinatal causes and there were seven (3%) congenital abnormalities, with no effect of in utero tenofovir exposure (*p*>0.4). Of 219 surviving infants, 182 (83%) enrolled in the follow-up study; median (interquartile range [IQR]) age at last visit was 25 (12–38) months. From mothers' ART, 62/9/111 infants had no/20%–89%/≥90% in utero tenofovir exposure; most were also zidovudine/lamivudine exposed. All 172 infants tested were HIV-negative (ten untested). Only 73/182(40%) infants were breast-fed for median 94 (IQR 75–212) days. Overall, 14 infants died at median (IQR) age 9 (3–23) months, giving 5% 12-month mortality; six of 14 were HIV-uninfected; eight untested infants died of respiratory infection (three), sepsis (two), burns (one), measles (one), unknown (one). During follow-up, no bone fractures were reported to have occurred; 12/368 creatinines and seven out of 305 phosphates were grade one (16) or two (three) in 14 children with no effect of in utero tenofovir (*p*>0.1). There was no evidence that in utero tenofovir affected growth after 2 years (*p* = 0.38). Attained height- and weight for age were similar to general (HIV-uninfected) Ugandan populations. Study limitations included relatively small size and lack of randomisation to maternal ART regimens.

**Conclusions:**

Overall 1-year 5% infant mortality was similar to the 2%–4% post-neonatal mortality observed in this region. No increase in congenital, renal, or growth abnormalities was observed with in utero tenofovir exposure. Although some infants died untested, absence of recorded HIV infection with combination ART in pregnancy is encouraging. Detailed safety of tenofovir for pre-exposure prophylaxis will need confirmation from longer term follow-up of larger numbers of exposed children.

**Trial registration:**

www.controlled-trials.com ISRCTN13968779

*Please see later in the article for the Editors' Summary*

## Abstract 2

Diana Gibb and colleagues investigate the effect of in utero tenofovir exposure by analyzing the pregnancy and infant outcomes of HIV-infected women enrolled in the DART trial.

## Editors' Summary

**Background:**

Currently, about 34 million people (mostly in low- and middle-income countries) are infected with HIV, the virus that causes AIDS. At the beginning of the epidemic, more men than women were infected with HIV but now about half of all people living with HIV/AIDS are women, most of who became infected through unprotected sex with an infected partner. In sub-Saharan Africa alone, 12 million women are HIV-positive. Worldwide, HIV/AIDS is the leading cause of death among women of child-bearing age. Moreover, most of the 400,000 children who become infected with HIV every year acquire the virus from their mother during pregnancy or birth, or through breastfeeding, so-called mother-to-child transmission (MTCT). Combination antiretroviral therapy (ART)—treatment with cocktails of powerful antiretroviral drugs—reduces HIV-related illness and death among women, and ART given to HIV-positive mothers during pregnancy and delivery and to their newborn babies greatly reduces MTCT.

**Why Was This Study Done?:**

Because of ongoing international efforts to increase ART coverage, more HIV-positive women in Africa have access to ART now than ever before. However, little is known about pregnancy outcomes among HIV-infected African women taking ART throughout pregnancy for their own health or about the long-term outcomes of their offspring. In particular, few studies have examined the effect of taking tenofovir (an antiretroviral drug that is now recommended as part of first-line ART) throughout pregnancy. Tenofovir readily crosses from mother to child during pregnancy and, in animal experiments, high doses of tenofovir given during pregnancy caused bone demineralization (which weakens bones), kidney problems, and impaired growth among offspring. In this study, the researchers analyze data collected on pregnancy and infant outcomes among Ugandan and Zimbabwean HIV-positive women who took ART throughout pregnancy in the Development of AntiRetroviral Therapy in Africa (DART) trial. This trial was designed to test whether ART could be safely and effectively delivered in Africa without access to the expensive laboratory tests that are routinely used to monitor ART toxicity and efficacy in developed countries.

**What Did the Researchers Do and Find?:**

The pregnancy outcomes of 302 women who became pregnant during the DART trial and information on birth defects among their babies were collected as part of the DART protocol; information on the survival, growth, and development of the infants born to these women was collected in a separate infant study. Most of the women who became pregnant were taking tenofovir-containing ART before and throughout their pregnancies. 58% of the pregnancies resulted in a live birth, 7% resulted in a stillbirth (birth of a dead baby at any time from 22 weeks gestation to the end of pregnancy), and 35% resulted in a termination or miscarriage (before 22 weeks gestation). Of the 226 live births, seven infants died within 2 weeks and seven had birth defects. Similar proportions of the infants exposed and not exposed to tenofovir during pregnancy died soon after birth or had birth defects. Of the 182 surviving infants who were enrolled in the infant study, 14 subsequently died at an average age of 9 months, giving a 1-year mortality of 5%. None of the surviving children who were tested (172 infants) were HIV infected. No bone fractures or major kidney problems occurred during follow-up and prebirth exposure to tenofovir in utero had no effect on growth or weight gain at 2 years (in contrast to a previous US study).

**What Do These Findings Mean?:**

By showing that prebirth tenofovir exposure does not affect pregnancy outcomes or increase birth defects, growth abnormalities, or kidney problems, these findings support the use of tenofovir-containing ART during pregnancy among HIV-positive African women, and suggest that it could also be used to prevent women of child-bearing age acquiring HIV-infection heterosexually. Notably, the observed 5% 1-year infant mortality is similar to the 2%–4% infant mortality normally seen in the region. The absence of HIV infection among the infants born to the DART participants is also encouraging. However, this is a small study (only 111 infants were exposed to tenofovir throughout pregnancy) and women were not randomly assigned to receive tenofovir-containing ART. Consequently, more studies are needed to confirm that tenofovir exposure during pregnancy does not affect pregnancy outcomes or have any long-term effects on infants. Such studies are essential because the use of tenofovir as a treatment for women who are HIV-positive is likely to increase and tenofovir may also be used in the future to prevent HIV acquisition in HIV-uninfected women.

**Additional Information:**

Please access these Web sites via the online version of this summary at http://dx.doi.org/10.1371/journal.pmed.1001217.

- Information is available from the US National Institute of Allergy and infectious diseases on all aspects of HIV infection and AIDS
- NAM/aidsmap provides basic information about HIV/AIDS, and summaries of recent research findings on HIV care and treatment (in several languages)
- Information is available from Avert, an international AIDS nonprofit on many aspects of HIV/AIDS, including detailed information on HIV/AIDS treatment and care, women, HIV and AIDS, children, HIV and AIDS, and on HIV/AIDS and pregnancy (some information in English and Spanish); personal stories of women living with HIV are available
- More information about the DART trial is available
- Additional patient stories about living with HIV/AIDS are available through the nonprofit website Healthtalkonline

## Introduction

Combination antiretroviral therapy (ART) is highly effective in reducing maternal mortality, morbidity, and mother-to-child transmission (MTCT) of HIV [1], and also allows for safe breast-feeding [2]. However there are few systematic prospectively collected long-term data on pregnancy rates and outcomes among HIV-infected African women taking combination ART throughout pregnancy for their own health or on long-term outcomes for their exposed children. Tenofovir DF is recommended as first-line ART in World Health Organization (WHO) 2010 guidelines [3]; its use has increased from 8% in December 2009 [4] to 19% by December 2010 [5] and is expected to further increase. In women of child-bearing age, future use may include pre-exposure prophylaxis; although the FEMPREP study evaluating its effectiveness in preventing HIV acquisition from sex between men and women was closed for futility, a more recent study found that tenofovir-containing regimens are highly effective in preventing HIV acquisition through heterosexual sex [6], similarly to those exposed through sex between men [7]. Whilst data on tenofovir use in pregnancy continue to accumulate [8], it remains a pregnancy category B drug, indicating that more information is needed on exposed mothers and infants [9]. This is particularly important given concerns about potential impact on bone mineralisation and renal impairment, delaying licensing for children, and the high rates of pregnancy in many African countries [10]–[13].

The Development of AntiRetroviral Therapy in Africa (DART) trial was designed to investigate the impact of routine laboratory monitoring for ART toxicity (haematology/biochemistry) and efficacy (CD4 counts) on long-term clinical outcomes compared to clinically driven monitoring (toxicity tests only if clinically indicated and no CD4 counts) [14]. 65% of trial participants were women, and three different initial ART regimens were used to increase generalisability. During the trial period (2003–2009), the majority of women who became pregnant were taking tenofovir-containing combination ART before and throughout pregnancy [11], providing an important opportunity to study pregnancy outcomes (secondary objective) and infant outcomes (primary objective). For the latter, a separate observational cohort substudy was designed to follow infants born to DART mothers 2004–2009 and describe growth and development, rates of HIV infection, and any adverse events (clinical, haematological, biochemical, and bone related) among those exposed and not exposed to tenofovir in utero.

## Methods

### Study Design, Setting, Population, and Maternal Follow-Up

**Figure 1:**
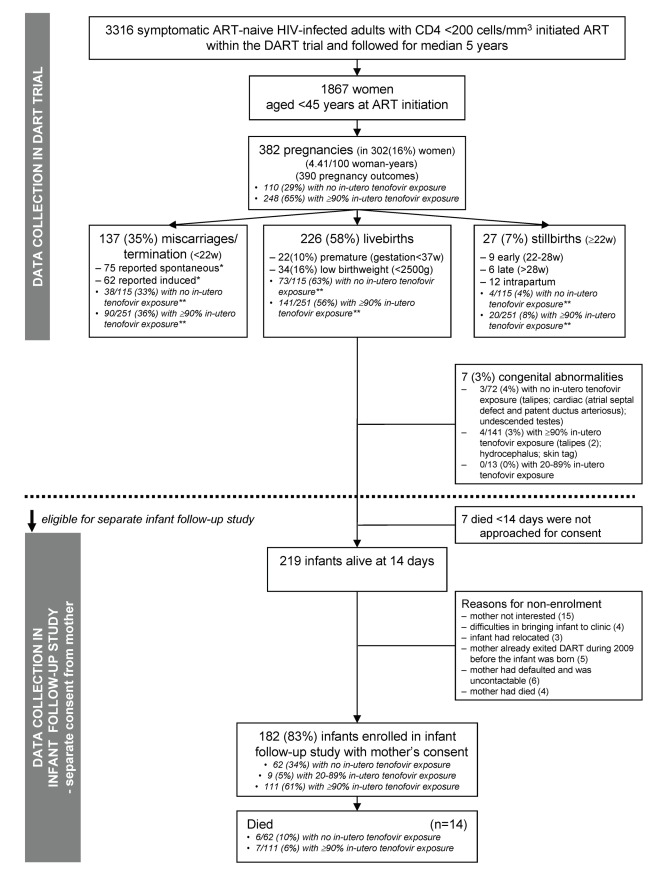
Flow diagram. *, Medical abortion is not generally legal in either Uganda or Zimbabwe. **, More pregnancies on tenofovir occurred in the first 3 y after ART initiation when the proportion of pregnancies ending in miscarriage/termination and stillbirth were higher and rates of live-births were lower.

2,156 antiretroviral-naive HIV-infected women with CD4 cell counts <200 cells/mm^3^ were enrolled in the DART trial (Uganda: Medical Research Council/Uganda Virus Research Institute [UVRI] Uganda Research Unit on AIDS, Entebbe; Joint Clinical Research Centre [JCRC], Kampala; and Infectious Diseases Institute [IDI], Mulago; Zimbabwe: University of Zimbabwe, Harare) from 15 January 2003–28 October 2004 [14], of whom 1,867 (87%) were <45 y old (Figure 1). DART was an open randomized trial comparing two management strategies for monitoring ART; routine laboratory and clinical monitoring (12 weekly CD4 and haematology/biochemistry tests) versus clinically driven monitoring (haematology/biochemistry tests only if clinically indicated). Women all had negative pregnancy tests at enrolment and started ART with zidovudine/lamivudine plus either tenofovir (in 2003/2004), or nevirapine or abacavir (2004 only) following WHO guidelines [15]. 369 of the 2,156 women in DART were randomised to receive nevirapine OR abacavir containing first-line ART in a nested randomised placebo-controlled toxicity substudy (NORA) in Uganda (placebo-controlled for 24 wk only; then open-label) [16]; remaining participants received tenofovir- or nevirapine-containing first-line ART (open-label and non-randomized). The only factor influencing choice of first-line ART was calendar year. During follow-up, women attended clinic four-weekly and had six-monthly pregnancy tests; those who became pregnant at any time were encouraged to seek immediate advice. Information on all pregnancies and pregnancy outcomes, including gestational age estimation based on last menstrual period, congenital abnormalities, and ART taken by the mother throughout pregnancy and the infant at birth were collected within DART. High levels of adherence to ART were reported in DART using pharmacy refill, pill counts, and questionnaires [14].

Outcome of pregnancy was categorised into: miscarriage or induced termination (<22 wk); early (22–28 wk), late (>28 wk), and intrapartum stillbirth; and live-birth. Low birth weight was defined as <2,500 g in an infant ≥37 wk. Prematurity was defined as <37 wk gestation.

Additional information on infants born to DART women was collected through a separate longitudinal observational study at all centres. Inclusion criteria were informed consent from the mother and willingness to comply with follow-up visits. Mothers whose child had died after the perinatal period were approached for consent to provide retrospective information. Infants born before November 2007 (Harare) or January–March 2008 (Uganda sites) were enrolled retrospectively and then followed prospectively; subsequent infants were prospectively enrolled to September 2009.

### Data Sources and Infant Follow-up

Infants were followed at 2, 6, 12, 24 wk, and then 24-weekly after birth until the mother exited DART (last information on pregnancy outcome December 2009). For infants enrolled retrospectively, data were extracted from medical records held at DART centres, where infants were already receiving regular follow-up care before ethics approval was obtained.

Clinical status, adverse events, infant feeding, weight, head, and mid-upper-arm circumference (MUAC), and growth were ascertained, and a neurodevelopment screen (evaluating gross/fine motor skills, hearing, understanding, expressive language, and social development, based on Bayley [17]) was undertaken at each visit. Routine biochemistry (creatinine, urea, liver enzymes [AST/ALT], sodium, potassium) and haematology (haemoglobin, MCV, WBC, lymphocytes, neutrophils, platelets) panels were performed in addition to urine dipsticks for protein and glucose, and blood calcium, phosphate, and alkaline phosphatase tests where samples were sufficient. The focus was on potential toxicities of tenofovir (creatinine, phosphate, alkaline phosphatase, urine tests) and cotrimoxazole or zidovudine (haemoglobin and neutrophils). Division of AIDS (DAIDS; US National Institutes of Health [NIH]) toxicity tables were used, with grades 1, 2, 3, and 4 defining mild, moderate, severe, and life-threatening events [18]; as neutrophils are lower in uninfected Africans, these used updated NIH paediatric toxicity tables [19]. For toxicity grading, infants with gestational age ≥37 wk were assumed to be born at “term.”

For infants enrolled prospectively at birth, a DNA or RNA PCR HIV test was performed at 6 wk and 12 mo. For breast-fed infants or where breast-feeding status was unknown, this was also done at 6 mo (or end of breast-feeding). For infants enrolled after birth, a PCR or HIV antibody test was done for those <18 or ≥18 mo, respectively, at first visit. All positive PCR tests were confirmed with a second test.

### Statistical Analysis

Data were analysed using Stata v11.0 (Stata Corporation). Analysis of pregnancy data collected in DART was an observational additional analysis, i.e., not by randomized group. In all analyses, pregnancies were considered statistically independent, because mothers were typically taking different ART in different pregnancies, and because, as subsequent pregnancies were only 21% of total, any variance adjustment would have little impact (results were similar restricting to the first pregnancy per woman). For similar reasons as only 3% infants were a twin, and because discordant HIV transmission among twins is well described, multiple births to the same mother were considered statistically independent in infant analyses (results were similar restricting to one enrolled infant per pregnancy). Generalised estimating equations with independent correlation structure were used to compare adherence during pregnancy with before/after pregnancy. Wilcoxon rank-sum tests were used to compare continuous variables between infants with no versus ≥90% in utero tenofovir exposure (tenofovir prescribed for ≥90% d from the estimated start to end of pregnancy); chi-squared and exact tests compared categorical variables. The 90% cut-off was arbitrary but chosen in advance of data analysis; it allowed 111 infants with ≥90% exposure to be compared with 62 infants with no in utero tenofovir exposure, excluding nine (5%) with 20%–89% exposure from tenofovir comparisons (see Table 1 for other components of the ART regimen).

**Table 1:** Characteristics for all enrolled infants and according to whether they had received no or ≥90% in utero exposure to tenofovir.

Characteristics	All Infants *n = *182	In Utero Tenofovir Exposure (*n = *173)^a^		
		0%, *n = *62	≥90%, *n = *111	*p*-Value
Female (%)	89 (49%)	29 (47%)	56 (50%)	0.64
**Gestational age (wk),** median (IQR)	39 (38–40)	39 (38–40)	39 (38–40)	0.79
28–<34	6 (3%)	4 (6%)	1 (1%)	
34–<37	11 (6%)	4 (6%)	7 (6%)	
37–<39	55 (30%)	15 (24%)	39 (35%)	
≥39	110 (61%)	39 (63%)	64 (58%)	
**Birth weight (kg),** median (IQR)	3.0 (2.5–3.2)	3.0 (2.6–3.3)	3.0 (2.5–3.2)	0.99
1–<2	16 (9%)	8 (13%)	6 (9%)	
2–<3	92 (54%)	28 (47%)	63 (61%)	
3–4.6	62 (36%)	24 (40%)	35 (34%)	
Not known	12	2	7	
**Mode of delivery**				0.41
Vaginal	145 (81%)	46 (75%)	91 (83%)	
Emergency caesarean	17 (9%)	8 (13%)	8 (7%)	
Elective caesarean	18 (10%)	7 (12%)	11 (10%)	
Not known	2	1	1	
**Ever breast fed,** *n* (%)	73 (40%)	26 (42%)	44 (40%)	0.77
**In utero maternal exposure to ART**				NA
100% first-line ART: zidovudine/lamivudine plus				
*Tenofovir*	99 (54%)	0 (0%)	94 (85%)	
*-With interruptions (3–12 wk)*	*10*	*0*	*5*	
*Abacavir*	6 (3%)	6 (10%)	0 (0%)	
*Nevirapine*	43 (24%)	43 (69%)	0 (0%)	
*-With interruptions (3–11 wk)*	*3*	*3*	*0*	
100% stavudine/lamivudine and a third drug^b^	13 (7%)	2 (3%)	11 (10%)	
100% lopinavir/ritonavir and two or three other drugs^c^	4 (2%)	3 (5%)	1 (1%)	
100% lamivudine/abacavir/nevirapine	1 (0.5%)	1 (2%)	0 (0%)	
changed one or more drugs during pregnancy^d^	16 (8%)	7 (11%)	5 (5%)	
**Infant exposure to antiretrovirals at and following delivery**				0.04
None	30 (16%)	11 (18%)	18 (16%)	
Single dose nevirapine	67 (37%)	17 (27%)	46 (41%)	
Single dose nevirapine + zidovudine (1–2 wk)	32 (18%)	18 (29%)	13 (12%)	
Single dose nevirapine + single dose zidovudine	5 (3%)	2 (3%)	3 (3%)	
Zidovudine (1 wk)	29 (16%)	11 (18%)	17 (15%)	
Other^e^	19 (10%)	3 (5%)	14 (13%)	
**Mother's CD4 cell count (cells per µl) at delivery (** ***n = *** **176 pregnancies)** f median (IQR)	293 (197–398)	296 (206–397)	278 (190–419)	0.44
<199	43 (26%)	13 (23%)	28 (26%)	0.50
200–349	68 (40%)	19 (34%)	45 (43%)	
350–499	41 (24%)	17 (30%)	22 (21%)	
500+	18 (10%)	7 (13%)	11 (10%)	
Not available	6	2	3	
**Mother's CD4 cell count (cells per µl) at ART initiation (** ***n = *** **176 pregnancies** g **)** median (IQR)	98 (34–137)	83 (18–129)	100 (48–141)	0.14
**Age at infant enrolment,** median (IQR) mo	12 (2–25)	10 (2–24)	13 (2–24)	0.41
<1	30 (17%)	11 (18%)	17 (15%)	
1–2	23 (13%)	8 (13%)	15 (14%)	
3–11	37 (20%)	17 (27%)	20 (18%)	
12–23	43 (23%)	11 (18%)	31 (28%)	
≥24	49 (27%)	15 (24%)	28 (25%)	
**Age at last visit,** median (IQR) mo	25 (12–38)	22 (13–36)	26 (12–38)	0.63
<1	12 (7%)	5 (8%)	6 (5%)	
1–2	32 (18%)	9 (14%)	22 (20%)	
3–11	44 (24%)	21 (34%)	23 (21%)	
24–35	42 (23%)	11 (18%)	30 (27%)	
≥36	52 (28%)	16 (26%)	30 (27%)	

*p*-Values from Wilcoxon rank sum tests (continuous) or chi-squared tests (categorical).

a Excludes *n = *9 with 22%–89% in utero exposure to tenofovir from comparison.

b One regimen with abacavir, one with nevirapine, and 11 with tenofovir.

c Lopinavir/ritonavir plus lamivudine/tenofovir/nevirapine (one), didanosine/nevirapine (one), didanosine/abacavir (two).

d Five switched to second line 9–21 wk into pregnancy (one of whom also had multiple substitutions on first and second line due to hypersensitivity and wrong dispensing); five had first-line substitutions (efavirenz to nevirapine [one] and stavudine to zidovudine [three] plus lamivudine/tenofovir; stavudine to lopinavir/ritonavir [for lipoatrophy]); four had second-line substitutions from efavirenz to nevirapine (two with lamivudine/lopinavir/ritonavir, two didanosine/lopinavir/ritonavir); and two intensified boosted protease inhibitor monotherapy (one with zidovudine/lamivudine/tenofovir for 33 wk, one with nevirapine for 15 wk of the pregnancy).

e Nevirapine single dose + zidovudine no length given (one), nevirapine only 1 wk (seven), nevirapine 1 wk + zidovudine 1 wk (one), zidovudine only 2–4 wk (four), zidovudine single dose (one), zidovudine and lamivudine 1 wk (three), didanosine 1 wk (one), stavudine 1 week (one).

f Closest within 9 wk before (*n = *76; median 20 d; IQR 12–30) or after (*n = *94; median 25 d; IQR 14–35) delivery for 170/176 pregnancies.

g 176 pregnancies in 152 mothers: median (IQR) pre-ART CD4 per mother 100 (34–145); *p* = 0.12 no versus ≥90% tenofovir.

NA, not applicable.

Survival was estimated using Kaplan-Meier methods. Time-dependent Cox proportional hazards models with analysis time from birth to death or last visit were used to examine the effects of feeding practice (currently breast-feeding versus previously breast-fed versus never breast-fed) on infant mortality. Missing dates were estimated as the mid-point between the last visit where current breast-feeding was recorded and the first visit recording no breast-feeding (*n = *18), or, if breast-feeding had already stopped, as the date of the first prospective visit (*n = *1). Infants with no stop reported were assumed to be still breast-feeding at the last visit (*n = *6, median 2 mo old, range 1–31).

The effects of no versus ≥90% in utero exposure to tenofovir on growth were examined using random effects models to allow for multiple measurements per child. z-Scores were calculated using “WHO Child Growth for 0–5 year olds” guidelines [20]; these relate observed growth parameters (height, weight, MUAC, and head circumference) to those expected in normal children according to percentiles. Thus a z-score of 0 indicates expected growth for age, and −/+1.96 indicates that growth is at the 2.5th or 97.5th percentile of that expected for age respectively. z-Scores <−6 (indicating severe impairment) were truncated at −6 (12 measurements only). Separate random effects models were fitted for four growth parameters: weight and head circumference z-scores from birth, MUAC z-scores from 12 wk, and height z-scores from 48 wk (due to measurement difficulties before this age). Changes in the speed of growth from birth (non-linearity) were investigated by profile log-likelihoods allowing two different initial and subsequent growth rates, changing at successive visit weeks; the maximum log-likelihood was used to estimate the timepoint where growth rate changed that was best supported by the data. Random effects were included for baseline value, initial, and subsequent slopes with an unstructured covariance matrix (except for MUAC and height where only overall slope random effects were used due to non-convergence of the full model).

### Ethical Considerations

Both the DART trial and the separate infant follow-up study (with separate protocol) were approved by Ethics Committees in Uganda, Zimbabwe and the UK.

## Results

1,867 women aged <45 y initiated ART in DART. During median follow-up of 5.1 y, there were 382 pregnancies in 302 (16%) women (Figure 1), an incidence of 4.4/00 woman-years (95% CI 4.0–4.9). 357 (93%) pregnancies occurred on first-line ART. As expected, pregnancy rates were highest in younger women (9.9 [8.6–11.5], 5.2 [4.4–6.1], and 1.8 [1.4–2.4] per 100 woman-years in women aged 18–29, 30–34, and 35–39 y at ART initiation, respectively; *p*<0.0001 adjusted for pre-ART CD4, centre, and years on ART). Pregnancy rates were also higher in rural Entebbe (adjusted risk ratio = 1.35 versus the main urban Kampala centre [48 km away] [1.06–1.73]; *p* = 0.02).

The 382 pregnancies resulted in 390 outcomes (including multiple births): 226 (58%) live-births, 137 (35%) miscarriages/terminations (75 [55%] reported as spontaneous, 62 [45%] as induced), and 27 (7%) stillbirths (Figure 1). There was marginal evidence of a decreasing proportion of miscarriages/terminations and stillbirths with increasing time since ART initiation (*p* = 0.06). There was no significant difference in the distribution of miscarriages/terminations, stillbirths, and live-births between women not on tenofovir versus those on tenofovir for ≥90% of their pregnancies (Figure 1, exact *p* = 0.19). 9.5% of 2,511 four-weekly adherence questionnaires completed during pregnancy reported missing one or more ART doses in the last month, compared to 9.9% in 16,246 questionnaires from the same women whilst they were not pregnant (*p* = 0.79). Four women died, two during pregnancy (severe malaria, septic abortion) and two at delivery (post partum haemorrhage, unknown cause).

The 226 live infants born between June 2004 and December 2009 were eligible for inclusion in the infant follow-up study (Figure 1). There were seven out of 226 (3%) live-births with congenital abnormalities: three out of 72 (4%) with no exposure versus four out of 141 (3%) with ≥90% in utero tenofovir exposure (exact *p* = 0.69) (Figure 1). (None of the stillbirths had congenital abnormalities reported). 22/225 (10%) live-births were premature (gestational age <37 wk) (one missing), eight of 72 (11%) with no tenofovir in utero exposure versus 13/140 (9%) with ≥90% exposure (chi-squared *p* = 0.67). Rates of low birth weight (<2,500 g) were 16% (34/209) among all live-births with birth weight recorded and 13% (25/189) among live-births with gestational age ≥37 wk.13/69 (19%) with no in utero tenofovir exposure had low birth weight compared with 19/130 (15%) with ≥90% in utero tenofovir exposure (chi-squared *p* = 0.44).

Seven mothers of live-births were not approached for consent. Their babies all died before 2 wk of age (six in the first day of life) of foetal distress (three), prematurity (three), and haemorrhagic disease (one); 1% (1/72) had no in utero tenofovir exposure versus 4% (six of 141) with ≥90% exposure (exact *p* = 0.40). Of 219 remaining eligible infants alive after 2 wk, 182 (83%) born to 152 mothers were enrolled in the follow-up study, including six pairs of twins, and 24 second or third births (see Figure 1 for reasons for non-enrolment). All 182 enrolled infants are included in further analyses: 129 (71%) were enrolled retrospectively (including some infants who had died), but clinical notes documenting regular follow-up were available for data collection (see Table 1 for age at enrolment). In utero tenofovir exposure was similar among 182 included and 37 excluded infants, as was centre, antiretrovirals received at birth, gestational age, and mode of delivery (*p>*0.22), although birth weight was lower for included infants (median 3 kg [interquartile range (IQR) 2.5–3.2]) compared to those excluded (3.2 kg [3–3.6]; *p* = 0.002).

### Baseline Characteristics and In Utero ART Exposure

62/182 (34%) infants had no in utero tenofovir exposure and 111 (61%) had ≥90% exposure; nine (5%) had 20%–89% exposure (excluded from tenofovir comparisons) (Table 1). 145 (81%) were delivered vaginally. Characteristics did not differ by in utero tenofovir exposure (*p>*0.4) (Table 1).

93% women (163/176, six twins) took ART throughout pregnancy, with only 13/176 (7%) interrupting ART for >7 d; infants were most commonly exposed in utero to zidovudine and lamivudine with tenofovir (54%) or nevirapine (24%). Overall 162/176 (92%) and nine out of 176 (5%) women were on first- and second-line ART regimens, respectively, and five (3%) switched from first- to second-line during pregnancy (Table 1). 152 (84%) infants received anti-HIV prophylaxis, the majority with single-dose nevirapine with or without additional short-course zidovudine. Compared to those with no tenofovir exposure, more infants with ≥90% in utero tenofovir exposure received single-dose nevirapine (as recommended in the DART procedures because this was a new drug their mothers were not taking).

The median age of infants at last visit was 25 (IQR 12–38) mo. Infants spent a median of 12 mo (IQR 5–17) in the study with median three (IQR 2–4) prospective clinic visits.

### HIV Testing and Transmission

172 of the 182 enrolled infants had HIV tests during the study: all were negative (latest HIV antibody and DNA PCR negative in 102 children aged ≥18 mo and 70 aged ≤18 mo, respectively). Two infants were lost to follow-up and eight died before being tested. The observed HIV transmission rate of 0% has an upper one-sided 97.5% confidence limit of 2.1% excluding those not tested. Sensitivity analyses assuming 100%, 50%, or 0% of the ten untested infants were infected gave transmission rates of 5.4% (95% CI 2.7%–9.9%), 2.7% (0.9%–6.3%), and 0.0% (97.5% one-sided CI 0.0%–2.0%), respectively.

The ten untested infants were last seen at median age 12 mo (range 2–34 mo); four died without a prospective follow-up visit, four died after one to six follow-up visits, and two were lost after two to three visits. Three were hospitalised for malaria, severe burns leading to death, and respiratory infection leading to death, respectively. Last weight-for-age z-scores (available for seven out of ten) were all above −2 except for one (−3.5). Neurodevelopment of untested children was described as normal (in clinic notes and according to more detailed questions on clinical case report forms) in all but one infant who at last follow-up at 24 wk could not sit unsupported.

### Mortality

14/182 infants died at median (IQR) age 9 (3–23) mo, giving 6- and 12-mo post-natal mortality of 3% and 5%, respectively (four per 100 child-years). Six infants were HIV negative and died of acute diarrhoea (two), malaria (one), airway obstruction (one), severe anaemia (one), and fever (one); eight HIV-untested infants died of respiratory infection (three), sepsis (two), burns (one), measles (one), and unknown cause of death (one). Four out of 14 (29%) were ever breast-fed. The mortality rate in the retrospective follow-up period was three per 100 child-years (95% CI 1–7) compared to four per 100 (95% CI 2–9) in the prospective period (*p* = 0.67). Six out of 62 (10%) with no in utero tenofovir versus seven out of 111 (6%) with ≥90% exposure died (*p* = 0.42) (one additional death had received 76% in utero tenofovir).

### Breast-feeding

In contrast to high breast-feeding rates generally observed in these settings, only 73 (40%) infants were ever breast-fed, for median 94 d (IQR 75–212): 88 (53–101) versus 144 (83–320) d in those with no tenofovir versus ≥90% exposure respectively (*p* = 0.03); only 24 (33%) infants were breast-fed for >6 mo. The unadjusted hazard ratio for mortality was 0.43 (95% CI 0.05–3.50) for currently breast-fed versus never-breast-fed babies and 0.70 (95% CI 0.19–2.60) for those who had stopped breast-feeding versus never-breast-fed (*p* = 0.68). Similar results were obtained adjusting for sex, gestational age, mode of delivery, and birth weight (unpublished data).

### Adverse Events, Clinical Events, and Medication

Among 368 serum creatinine measurements in 164 children, only 12 (3%) were abnormal (three grade 2, nine grade 1), and hypophosphataemia occurred in only seven out of 305 (2%) samples (all grade 1); no differences were observed between children with no tenofovir exposure versus ≥90% in utero exposure (*p>*0.15) (Table 2). Consecutive creatinine or phosphate values were abnormal in only two out of 14 children, neither of whom were exposed to tenofovir in utero. Five out of 109 children had a single occurrence of 1+ proteinuria: one had no in utero tenofovir and four had ≥90% exposure. All 112 sugar dipsticks were negative. Low haemoglobin (*n = *95, 56%), mostly grade 1 (*n = *51) or grade 2 (*n = *26), occurred slightly more frequently in infants without in utero tenofovir exposure (*p* = 0.06), but in similar percentages of children with no zidovudine versus >90% in utero zidovudine exposure (14/24 [58%] versus 74/130 [57%]; *p* = 0.90). No bone fractures were reported to have occurred in any child.

**Table 2:** Creatinine, phosphate, and haemoglobin toxicity.

Parameter	Total	In Utero Tenofovir Exposure^a^		
		0%	≥90%	*p*-Value
**Creatinine**				
Number of measurements recorded	368	123	231	
Number with grade 1–4 toxicity	12	3	9	
Grade 1 (≥1.1–<1.4×uln)	9	2	7	
Grade 2 (≥1.4–<1.9×uln)	3	1	2	
Number of children with creatinine measured (percent of enrolled children)	164 (90%)	52 (84%)	99 (89%)	
Median (IQR) measurements per child	2 (1–3)	2 (1–3)	2 (1–3)	0.65
Children with grade 1–4 toxicity	11 (7%)	3 (6%)	8 (8%)	0.60
Maximum grade 1	8	2	6	
Maximum grade 2	3	1	2	
**Phosphate**				
Number of measurements recorded	305	106	186	
Number with grade 1–4 toxicity	7	5	1	
Grade 1^b^	7	5	1	
Number of children with phosphate measured (percent of enrolled children)	161 (88%)	57 (92%)	96 (86%)	
Median (IQR) measurements per child	2 (1–2)	2 (1–2)	1 (1–2)	0.57
Children with grade 1–4 toxicity	5 (3%)	3 (5%)	1 (1%)	0.15
Maximum grade 1	5	3	1	
**Alkaline phosphatase**				
Number of measurements recorded	364	122	228	
Number with grade 1–4 toxicity	44	15	29	
Grade 1 (>1.25–≤2.5×uln)	39	13	26	
Grade 2 (>2.5–≤5×uln)	3	1	2	
Grade 3 (>5–≤10×uln)	2	1	1	
Number of children with alkaline phosphatase measured (percent of enrolled children)	165 (91%)	58 (94%)	99 (89%)	
Median (IQR) measurements per child	2 (1–3)	2 (1–3)	2 (1–3)	0.63
Children with grade 1–4 toxicity	31 (19%)	11 (19%)	20 (20%)	0.85
Maximum grade 1	26	9	17	
Maximum grade 2	3	1	2	
Maximum grade 3	2	1	1	
**Haemoglobin**				
Number of measurements recorded	410	140	253	
Number with grade 1–4 toxicity	157 (38%)	61 (44%)	94 (37%)	
Grade 1^c^	93	33	58	
Grade 2	37	14	23	
Grade 3	22	10	12	
Grade 4	5	4	1	
Number of children with haemoglobin measured (percent of enrolled children)	169 (93%)	59 (95%)	102 (92%)	
Median (IQR) measurements per child	2 (1–3)	2 (1–3)	2 (1–3)	0.92
Children with grade 1–4 toxicity	95 (56%)	40 (68%)	54 (53%)	0.06
Maximum grade 1	51 (30%)	19 (32%)	31 (30%)	
Maximum grade 2	26 (15%)	12 (20%)	14 (14%)	
Maximum grade 3	13 (8%)	5 (8%)	8 (8%)	
Maximum grade 4	5 (3%)	4 (7%)	1 (1%)	
**Platelets**				
Number of measurements recorded	409	139	253	
Number with grade 1–4 toxicity	19	5	13	
Grade 1 (100–125×10^9^/l)	9	1	7	
Grade 2 (50–99×10^9^/l)	5	1	4	
Grade 3 (25–49×10^9^/l)	5	3	2	
Number of children with platelets measured (percent of enrolled children)	169 (93%)	59 (95%)	102 (92%)	
Median (IQR) measurements per child	2 (1–3)	2 (1–3)	2 (1–3)	0.86
Children with grade 1–4 toxicity	16 (9%)	5 (8%)	10 (10%)	0.78
Maximum grade 1	6	1	4	
Maximum grade 2	5	1	4	
Maximum grade 3	5	3	2	
**Neutrophils**				
Number of measurements recorded	409	139	253	
Number with grade 1–4 toxicity	18	6	12	
Grade 1^c^	12	3	9	
Grade 2	3	2	1	
Grade 3	3	2	2	
Number of children with neutrophils measured (percent of enrolled children)	169 (93%)	59 (95%)	102 (92%)	
Median (IQR) measurements per child	2 (1–3)	2 (1–3)	2 (1–3)	0.86
Children with grade 1–4 toxicity	18 (11%)	6 (10%)	12 (12%)	0.76
Maximum grade 1	12	3	9	
Maximum grade 2	3	2	1	
Maximum grade 3	3	2	1	

*p*-Values from Wilcoxon rank sum tests (continuous) or chi-squared tests (categorical; exact tests used where <5% in any cell). Consecutive creatinine or phosphate values were abnormal in only two out of 14 children, neither of whom were exposed to tenofovir in utero. Few other laboratory abnormalities were observed: alanine transaminase (ALT), 12/364 measured; aspartate transaminase (AST), 21/344; low sodium, 73/364; low potassium, one out of 360; low calcium, eight out of 389. All abnormalities were grade 1 apart from four grade 2 and one grade 4 hyponatraemia.

a Excludes *n = *9 with 22%–89% in utero exposure to tenofovir from comparison.

b Grade 1 phosphate is ≥3.5 to <4.5 mg/dl (≥1.13 to <1.45 mmol/l) under 1 y of age; and ≥3.0 to <3.5 mg/dl (≥0.97 to <1.13 mmol/l) over 1 y of age.

c See Table S1 for toxicity grades for haemoglobin and neutrophils in HIV-uninfected infants and children.

ULN, upper limit of normal.

51 children had one or more hospitalisations (total 85 hospitalisations, maximum six per child); incidence 22 hospitalisations (95% CI 18–28) per 100 child-years (31 [22]–[42] versus 18 [13]–[24] for those with none versus ≥90% in utero tenofovir exposure respectively; *p* = 0.02). Nearly half (40/85, 47%) were for malaria. Other events requiring hospitalisation included diarrhoea (14/85, 16%), pneumonia (ten out of 85, 12%), meningitis/sepsis (eight out of 85, 9%), and clinical anaemia (five out of 85, 6%), with no significant differences between those with none versus ≥90% in utero tenofovir exposure for any of these (*p*≥0.13). Of 77 additional clinical events not requiring hospitalisation, 66 (86%) were malaria and four (5%) were clinical anaemia. The unadjusted hazard ratio for time to first hospitalisation or death was 0.44 (95% CI 0.17–1.14) for currently breast-fed versus never-breast-fed babies and 0.90 (95% CI 0.50–1.66) for those who had stopped breast-feeding versus never-breast-fed (*p* = 0.19).

### Growth

**Figure 2:**
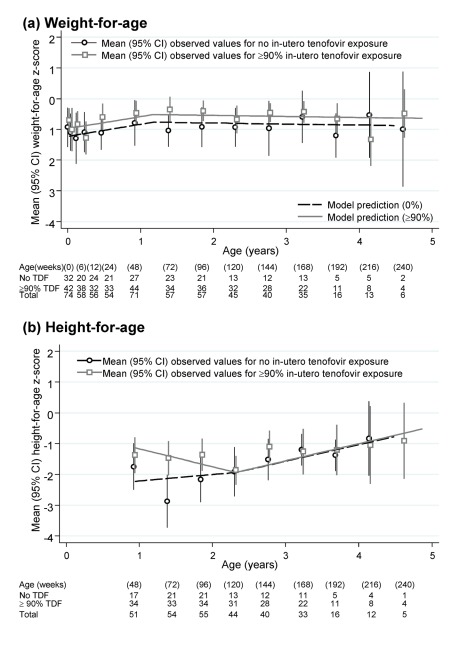
Height-for-age and weight-for-age z-scores in infants born to HIV-infected mothers taking ART. Numbers and mean (95% CI) based on the closest measurement per child to scheduled visit weeks, which were at 6, 12, 24 wk, and then 24-weekly.

Weight and height at birth were significantly lower than average according to WHO norms (both *p*<0.001) (Table 3). There was also marginal evidence of lower than expected MUAC at 12 wk (*p* = 0.06). After birth, weight, and MUAC z-scores increased significantly to age 60 wk, remaining stable (weight for age) or slightly decreasing (MUAC) thereafter. At 1.5 and 3 y of age, mean weight, MUAC, and head circumference z-scores were well within the normal range, with variability as expected in the WHO reference population. Predicted weights and MUAC were also similar to the median from a reference group of under 5-y-old children in Mbarara, south-west Uganda (Table 3) [21]. In contrast, height for age slightly decreased from week 48 to 120, followed by moderate increases from 120 wk onward (heterogeneity *p* = 0.004). The net effect of these changes was that children remained significantly stunted at 1.5 and 3 y according to WHO norms (mean z-scores −2.14 and −1.32, respectively), although predicted heights (in centimetres) were comparable to a Ugandan reference population [21]. We found no evidence of differences between children with no in utero tenofovir versus ≥90% exposure in weight (Figure 2a), MUAC, or head circumference for age, either initially (*p>*0.1) or subsequently (*p>*0.8). Height-for-age z-scores were in fact lower in those with no in utero tenofovir exposure before 2 y old (*p* = 0.03), but similar thereafter (*p* = 0.38) (Figure 2b). This appeared to be driven by somewhat greater height for age in those with ≥90% tenofovir exposure at 48 wk, which subsequently decreased slightly to match those with no in utero tenofovir exposure by 2 y. Head circumference z-score did not vary with age (*p* = 0.91).

**Table 3:** Changes in height, weight, head circumference, and MUAC z-scores with age in HIV-exposed infants.

Model/Factor	Weight for Age (Modelled from Birth onwards)	Head Circumference for Age (Modelled from Birth onwards)	MUAC for Age (Modelled from 12 wk Only onwards)	Height for Age (Modelled from 48 wk Only onwards)
**Overall model**				
Number of measurements (number of children, percent of enrolled children)	643 (178, 98%)	382 (154, 85%)	374 (157, 86%)	322 (144, 79%)
Median (IQR) per child	4 (2–5)	2 (1–3)	2 (1–3)	2 (1–3)
Mean (SD) at baseline^a^	−1.03 (1.62)	+0.24 (1.57)	−0.37 (2.47)	−1.51 (2.34)
Evidence for variation in trends over time (change point)	*p*<0.001 (at 60 wk)	*p* = 0.14 (at 60 wk)	*p*<0.001 (at 60 wk)	*p* = 0.004 (at 120 wk)
Mean (95% CI) change in z-score per year before changepoint	+0.40 (+0.13 to +0.67)	+0.009 (−0.16 to +0.17)	+1.00 (+0.55 to +1.46)	−0.27 (−0.57 to +0.02)
Mean (95% CI) change in z-score per year after changepoint	−0.03 (−0.16 to +0.09)	(as above)	−0.23 (−0.37 to −0.09)	+0.54 (+0.24 to +0.84)
Predicted^b^ mean z-score (SD)				
At 1.5 y old^a^	−0.59 (1.05)	+0.26 (0.87)	+0.75 (0.85)	−2.14 (1.32)
At 3 y old^a^	−0.63 (2.12)	+0.27 (1.10)	+0.41 (0.69)	−1.32 (1.02)
Predicted^b^ mean absolute value				
At 1.5 y old girl/boy	9.5/10.2 kg	46.6/47.7 cm	15.4/15.7 cm	74.5/76.5 cm
At 3 y old girl/boy	12.8/13.3 kg	48.9/49.8 cm	16.2/16.3 cm	90.0/91.4 cm
Median values in reference population in south-west Uganda [21]				
At 1.5 y old girl/boy	8.8/10.1 kg	N/A	14.4/14.7 cm	76.0/77.4 cm
At 3 y old girl/boy	12.8/13.0 kg	N/A	15.2/15.3 cm	88.9/89.3 cm
**Including in utero tenofovir exposure**				
Number of measurements (number of children)^c^	612 (169)	372 (149)	361 (151)	310 (138)
Mean (SD) at baseline				
No tenofovir in utero	−1.22 (2.16)	+0.07 (2.23)	−0.40 (2.82)	−2.22 (3.80)
≥90% tenofovir in utero	−0.97 (1.85)	+0.29 (1.79)	−0.37 (2.56)	−1.13 (2.84)
Mean (95% CI) change in z-score per year before changepoint^d^	(at 60 wk)		(at 60 wk)	(at 120 wk)
No tenofovir in utero	+0.36 (−0.08 to + 0.80)	+0.03 (−0.24 to +0.30)	+1.09 (+0.40 to +1.77)	+0.20 (−0.30 to +0.72)
≥90% tenofovir in utero	+0.42 (+0.07 to + 0.77)	+0.02 (−0.18 to +0.22)	+0.91 (+0.30 to +1.52)	−0.58 (−0.94 to −0.21)
Mean (95% CI) change in z-score per year after changepoint				
No tenofovir in utero	−0.02 (−0.25 to +0.20)	(as above)	−0.20 (−0.44 to +0.05)	+0.54 (−0.003 to +1.08)
≥90% tenofovir in utero	−0.04 (−0.21 to −0.13)	(as above)	−0.22 (−0.40 to −0.04)	+0.55 (+0.16 to +0.95)
Heterogeneity in time trends between infants exposed to tenofovir or not^e^	*p* = 0.98	*p* = 0.93	*p* = 0.89	*p* = 0.04^f^
Overall heterogeneity (including baseline) between infants exposed to tenofovir or not^g^	*p* = 0.55	*p* = 0.58	*p* = 0.96	*p* = 0.05^f^

Effects of tenofovir exposure on the different outcomes were similar adjusting for other factors at birth in Table 1; these cannot confound the relationship between growth and tenofovir exposure as there were no significant differences in these factors according to in utero tenofovir.

a Would expect mean (standard deviation [SD]) of 0 (one) in a normal population: baseline is birth for weight and head circumference for age, week 12 for MUAC for age, and week 48 for height for age.

b Predicted z-scores estimated from the fixed-effects population average fitted model at the ages above and back-transformed into sex-specific weights using the z-score formula.

c Median (IQR) per child with 0% or ≥90% in utero tenofovir exposure same as the whole group.

d Identical changepoints identified using profile log-likelihood as for the whole group.

e *p*-Value from a 2 degree of freedom (df) Wald test (1 df for head circumference) comparing time trends before and after changepoint.

f Driven by estimated small decline in height for age from 48–120 wk (and greater height for age at 48 wk) in those with ≥90% tenofovir in utero exposure (Figure 2): *p* = 0.38 comparing height-for-age trajectories after 120 wk.

g *p*-Value from a 3 df Wald test (2 df for head circumference).

## Discussion

The goals of our study were to provide long-term information on infants exposed to combination ART with and without tenofovir throughout intrauterine and breast-feeding periods as a primary objective; describing pregnancy outcomes among women taking tenofovir for their own health was a secondary objective. Since DART started in 2003, tenofovir has become a recommended drug in first-line ART regimens in adult guidelines worldwide [3],[22]–[24]. With new recommendations to start ART earlier [3],[23],[24], and evidence of reduced onward HIV transmission with earlier ART initiation [25], the potential that women may conceive and/or go through pregnancy while taking tenofovir is likely to increase. Furthermore, recent results demonstrating effectiveness of tenofovir in preventing HIV acquisition in serodiscordant heterosexual couples [6] mean that it is likely to have an increasing role in preventing HIV acquisition in HIV-uninfected women having sex with men. However, the reasons for lack of effect in another study [26] require more investigation. The fact that the same dose (300 mg daily) is used in PrEP and HIV treatment suggests our results should be generalisable to both indications.

Given advanced immunodeficiency (CD4<200 cells/mm^3^) at ART initiation in all included women, it is not surprising that we observed somewhat lower pregnancy rates than the 9.0/100 women-years on ART recently reported from the MTCT-Plus initiative [12]. The pregnancy rate of 4.4/100 woman-years in DART was more similar to the rate in HIV-infected women not on ART (6.5) in MTCT-Plus. However, our results suggest that, on average, at least one in 20 HIV-infected women on ART was having unprotected sex every year, and highlights the importance of improved contraception with counselling as women's health improves on ART. Our miscarriage/termination rate of 35% is higher than observed in settings without regular pregnancy testing; but it is similar to the 42% reported in the Botswana Tschepo trial [27]. Even higher rates (70%) were reported among HIV-infected women enrolling in microbicide preparedness studies [28]. Whilst around half the miscarriages/terminations were reported as spontaneous in our study, the distinction between induced termination and spontaneous termination/miscarriage is problematic in settings where medical termination is not legal, and may also not have been disclosed in all DART centres. Of note, we found no evidence that pregnancy outcome was related to use of tenofovir in pregnancy.

Tenofovir is present at low concentrations in breastmilk [29] but readily crosses the placenta in pregnant women on ART [30], thus exposing infants to relatively high levels in utero. Safety issues for tenofovir-exposed infants include concerns about congenital abnormalities, renal function, bone mineralisation, and growth. These concerns first arose from studies showing that high doses of tenofovir given to pregnant Rhesus macaques caused bone demineralisation, impaired somatic growth, and low levels of insulin-like growth factor-1 in their infants [31]–[33]. Renal tubular leak of phosphate resulted in bone demineralization and deformities in macaque infants given high dose tenofovir, although these effects were largely reversible once tenofovir was stopped [31].

Rates of congenital abnormalities in our study are similar for tenofovir and non-tenofovir exposed infants and to those from the Antiretroviral Pregnancy Register (APR), which recently reported a congenital anomaly prevalence among infants with first trimester exposure to tenofovir of 2.4% (26 of 1,092 live births), with no specific pattern of anomalies [8]. This is also similar to the congenital abnormality rate in the general population, for example, 2.7% reported in the US Centers for Disease Control's Congenital Defects Program 1989–2003 [34]. It is important that African data such as those from DART continue to also be reported to the APR, which monitors congenital abnormalities following exposure to all antiretroviral drugs. Prematurity occurring in pregnant women on ART has been attributed mainly to protease inhibitor (PI)-based ART [35]–[37]; in our study, where few women were on PIs, the prematurity rate of ∼10% did not differ by tenofovir exposure and is similar to rates reported in women either not on ART or on non-PI–based ART in these studies.

Despite increasing numbers of infants being exposed to tenofovir in utero and use of off-label tenofovir in HIV-infected children, data on its effects on renal function, bone mineralization, and growth remain relatively sparse; in particular, few studies have included a control group. Whilst our study was relatively small, we had >80% power to detect absolute differences of ∼20% between tenofovir-exposed and non-exposed infants. Although not randomized, we observed no evidence of increased renal impairment or hypophosphataemia among infants exposed to tenofovir- versus non-tenofovir–containing ART, in contrast to reports in older HIV-infected children [38],[39], including a case-control study that reported hypophosphataemia of grade 2 or greater in ∼4% UK/Irish children receiving off-label tenofovir versus ∼1% controls [40],[41]. Although abnormalities appear to normalise following withdrawal of tenofovir [39],[40], more long-term data are needed in children receiving tenofovir from an early age, including through adolescence. Despite best efforts, we failed to collect sufficient urine samples for calcium/phosphate measurements in our study and although there was no evidence of significant proteinuria, more sensitive markers of tubular dysfunction were not measured. An increased risk of fractures has been reported in HIV-infected versus HIV-uninfected adults [42],[43], and reduced bone density was one of few adverse effects observed more frequently in those continuing versus interrupting ART in the SMART trial [44]. Reductions in bone density with tenofovir have also been reported in some [45]–[47], but not all [48] studies. Fractures have not been reported systematically from studies of ART in children but appear to be uncommon; none were reported in our small study. More sophisticated dual-energy x-ray absorptiometry (DEXA) scanning is difficult in very young children, and is also limited by lack of normative data in African children and expense. Longer term data on bone health in both tenofovir-exposed HIV-infected children and infants exposed to in utero tenofovir are needed, particularly as tenofovir has recently been licensed by the US Food and Drug Administration for adolescents >12 y and is expected in younger children.

With respect to growth among tenofovir-exposed infants, Nurutdinova reported one of 14 live-births to tenofovir-exposed women to be small for gestational age [49]. The US Pediatric HIV/AIDS Cohort Study (PHACS) of 532 infants reported lower weight but not height at 1 y among those exposed versus not exposed to tenofovir in utero [50], although the effect was unrelated to duration of tenofovir exposure and was not present at birth. The authors concluded that tenofovir should remain an “alternative” rather than “recommended” drug during pregnancy as per current US guidelines [51]. We found no evidence of lower birth-weights or any subsequent growth parameter up to age 4 y among children exposed to tenofovir throughout intrauterine life compared with unexposed children, and had >80% power to detect differences of ∼0.5 z-score units. Powis et al. observed lower birth weights in triple (but non-tenofovir) ART compared with zidovudine-only exposed infants in a combined analysis of nearly 2,000 infants in the Botswanan Mma Bana and Mashi trials [52]; as in our study there was catch-up weight gain soon after birth, more so in the triple ART group. Despite somewhat lower height-for-age z-scores compared to WHO norms, both weights and heights attained among children in our study were very similar to those reported by Cortinovis et al. in a large reference study of over 4,000 young HIV-uninfected children in south-west Uganda [21], demonstrating the difficulties in assessing what is “normal” growth for children in different geographical locations.

While the focus of this study was on evaluating possible adverse effects of in utero exposure to tenofovir in infants, many were also exposed to zidovudine/lamivudine. Although low-grade anaemia was not uncommon, there was no evidence to suggest this occurred more frequently in those with in utero zidovudine exposure. Assessment of mitochondrial dysfunction and neurolodevelopmental outcomes is limited but no overt manifestations were observed.

In agreement with other studies of women receiving ART during pregnancy and breast-feeding [1],[2], the rate of pMTCT was low. Although we observed no HIV-infected infants, testing for HIV infection was not 100%. However, under the most pessimistic assumptions about the HIV prevalence in untested infants, the estimated pMTCT rate would still be only ∼5%, despite women having had very advanced disease (32%<50 cells/mm^3^ at ART initiation). Half the women in DART received ART with clinically driven laboratory toxicity monitoring only and no routine CD4 tests throughout pregnancy. Our results reassure that lack of monitoring tests should never be a barrier to providing ART to pregnant women in resource-limited settings to effectively prevent MTCT and for their own health. Of note, we purposely recommended that all infants receive single dose nevirapine prophylaxis to provide additional pMTCT “cover,” in case any mothers on zidovudine/lamivudine/tenofovir were viraemic at delivery.

Despite encouragement to breast-feed, only 40% women in this cohort did so, and then only for a short time. WHO guidelines at the time recommended that women make their own choices about infant feeding [53], and there was considerable pressure not to breast-feed; if women chose to breast-feed, early weaning was encouraged, particularly in Uganda (personal observation, P Musoke). Mortality in our cohort was 5% by age 12 mo (3.4% by 6 mo), not dissimilar to those of Powis et al. who reported mortality of 2.1% by 6 mo in infants of Botswanan women taking combination ART who all exclusively breast-fed for longer [52]. Whilst we did not find a significant association between mortality and formula- versus breast-fed infants, likely because of lack of power, the large effect estimate is in keeping with other African studies [54]–[56], which led to revision of WHO guidelines to encourage breast-feeding while providing antiretroviral prophylaxis either to the mother or the baby while breast-feeding [57], as occurred in DART. There was marginal evidence of fewer hospitalisations in children exposed to tenofovir in utero, although numbers were too few to identify whether or not breast-feeding might be the reason for this; there was certainly no evidence of excess morbidity associated with in utero tenofovir exposure.

The main limitation of both the primary outcomes in infants and secondary pregnancy outcomes in mothers is the relatively small sample size. However, data on mothers receiving combination ART for their own health throughout pregnancy in resource-limited settings remain scarce, although undoubtedly further important information will be gathered with changes in WHO guidelines to recommend combination ART for all pregnant women (Option B+) [3]. The main DART trial was not designed to investigate issues in pregnancy, and most ART was non-randomised—comparisons of tenofovir exposure are therefore observational rather than randomised and the possibility of bias cannot be excluded.

In conclusion, we observed no evidence that tenofovir versus non-tenofovir ART had any adverse effects on pregnancy outcomes or on congenital, renal, bone, or growth abnormalities up to age 4 y among children born to women with severe HIV immunodeficiency at ART initiation and exposed throughout the intrauterine period. Although some infants died untested for HIV, the estimated MTCT rate taking the most pessimistic scenario was ∼5% and no HIV-infected babies were found. Infant mortality was low and similar to the general population of 2%–4% for these African countries in 2010 [58]. Our findings suggest tenofovir-containing ART is a reasonable choice in pregnancy and that tenofovir pre-exposure prophylaxis is also reasonable for women who are at high risk of seroconverting during pregnancy [59],[60]; safety of tenofovir for PrEP will only be confirmed by longer term follow-up of large numbers of exposed infants.

## Supporting Information

Table S1 **Toxicity grades for haemoglobin and neutrophils in HIV-uninfected infants and children.**
(DOC)

Text S1 **Study protocol.** Follow-up of infants born to HIV-infected women in the DART study.
(PDF)

## Abbreviations

ART: antiretroviral therapy
DART: Development of AntiRetroviral Therapy in Africa
IQR: interquartile range
MTCT: mother-to-child transmission
MUAC: mid-upper-arm circumference
WHO: World Health Organization
