# Supplementary material for: Pregnancy and Infant Outcomes among HIV-Infected Women Taking Long-Term ART with and without Tenofovir in the DART Trial
Source: PLoS Med. 2012 May 15;9(5):e1001217. doi: 10.1371/journal.pmed.1001217 (PMC3352861; doi:10.1371/journal.pmed.1001217)
Supplement: Table S1 — Toxicity grades for haemoglobin and neutrophils in HIV-uninfected infants and children. (DOC) [file pmed.1001217.s001.doc]

**Table S1 - Toxicity grades for haemoglobin and neutrophils in HIV uninfected infants and children**

(a) Haemoglobin

| PARAMETER | GRADE 1 | | GRADE 2 | GRADE 3 | GRADE 4 |
| --- | --- | --- | --- | --- | --- |
| ≥ 57 days | | 10 – <11 g/dL | 9 – <10 g/dL | 7 – <9 g/dL | <7 g/dL |
| Infant, 36–56 days | | 8.5 – <9.5 g/dL | 7 – <8.5 g/dL | 6 – <7 g/dL | < 6 g/dL |
| Infant, 22–35 days | | 9.5 – <10.5 g/dL | 8 – <9.5 g/dL | 7 – <8 g/dL | < 7 g/dL |
| Infant, 1–21 days | | 12 – <13 g/dL | 10 – <12 g/dL | 9 – <10 g/dL | < 9 g/dL |

(b) Absolute neutrophils

| PARAMETER | GRADE 1 | GRADE 2 | GRADE 3 |
| --- | --- | --- | --- |
| > 7 days | 750 –< 1,000/mm^3^ | 500 – 749/mm^3^ | 250 – 499/mm^3^ |
| Infant, 2–7 days | 1,250 – 1,500/mm^3^ | 1,000 – 1,249/mm^3^ | 750 – 999/mm^3^ |
| Infant, 1 day | 4,000 – 5,000/mm^3^ | 3,000 – 3,999/mm^3^ | 1,500 – 2,999/mm^3^ |
