# Supplementary material for: Pregnancy and Infant Outcomes among HIV-Infected Women Taking Long-Term ART with and without Tenofovir in the DART Trial
Source: PLoS Med. 2012 May 15;9(5):e1001217. doi: 10.1371/journal.pmed.1001217 (PMC3352861; doi:10.1371/journal.pmed.1001217)
Supplement: Text S1 — Study protocol. Follow-up of infants born to HIV-infected women in the DART study. (PDF) [file pmed.1001217.s002.pdf]

DART

Development of **A**nti**R**etroviral **T**herapy
in Africa

Follow up of infants born to HIV infected women in the DART study

Protocol number 1.2

Protocol date 16/07/2008

Authorised by:

Name: Professor Ian Weller Role: Chair of DART TSC

....
Signature: Date:

General Information and Organisation

This document describes a longitudinal observational study conducted in conjunction with the **D**evelopment of **A**nti**R**etroviral **T**herapy in Africa (DART) study and provides information about procedures for enrolling children born to women in DART.

Main Contacts

- **Mulago Hospital/Academic Alliance, Mulago**

Dr Philippa Musoke	Tel:	+ 256 41 541 044
Department of Paediatrics	Email:	pmusoke@mujhu.org
PO Box 7072	Fax:	+ 256 41 541 044
Makerere University		
Kampala, Uganda		

Prof Addy Kekitiinwa	Tel:	+ 256 41 541 044
Makerere University Medical School	Fax:	+ 256 41 531 667
Paediatrics and Child Health, Mulago Hospital	Email:	akekitiinwa@pidc.idi.co.ug
7051 Kampala, Uganda		

- **Joint Clinical Research Centre, Kampala**

Dr Victor Musiime	Tel:	+ 256 41 270 283/622
Joint Clinical Research Centre	Fax:	+256 41 342632/271260
P O Box 10005	Email:	musiimev@yahoo.co.uk
Kampala, Uganda		

- **Pari Hospital, Harare**

Prof Kusum Nathoo	Tel:	+ 263 4 705 986
	Email:	knathoo@medsch.uz.ac.zw
Dr Mutsawashe Filda Dangarembizi	Tel:	+ 263 4 705 986
	Email:	mdanga@mweb.co.zw
Department of Paediatrics and Child Health	Fax:	+263 4 724 912
University of Zimbabwe		
Box A178, Avondale		
Harare, Zimbabwe		

- **Medical Research Council Programme on AIDS, Entebbe**

Dr Paula Munderi	Tel:	+ 256 41 321 461
Medical Research Council Programme on AIDS	Fax:	+ 256 41 321 137
c/o Uganda Virus Research Institute	E-mail:	paula.munderi@mrcuganda.org
P. O. Box 49		
Entebbe Uganda		

- **MRC Clinical Trials Unit, London**

Prof Diana Gibb	Tel:	+ 44 20 7670 4709
MRC Clinical Trials Unit	Fax:	+ 44 20 7670 4818
222 Euston Road	E-mail:	di.gibb@ctu.mrc.ac.uk
London NW1 2DA, UK		
Dr Hermione Lyall	Tel:	+ 44 20 7886 1013
St Mary's Hospital,	Fax:	+ 44 20 7886 6341
South Wharf Road, Paddington	Email:	hermione.lyall@st-marys.nhs.uk
London W2 1NY, UK		

DART & ARROW Project Leaders & Trial Managers

- **Medical Research Council Programme on AIDS, Entebbe**

Dr Paula Munderi (DART co-PI) Tel: + 256 41 321 461
Medical Research Council Programme on AIDS Fax: + 256 41 321 137
c/o Uganda Virus Research Institute E-mail: paula.munderi@mrcuganda.org
P. O. Box 49
Entebbe Uganda
Dr Patricia Nahirya (ARROW Project Leader) Email: Patricia.Nahirya@mrcuganda.org
Dr Geoffrey Kabuye (DART Trial Manager) Email: geoffrey.kabuye@mrcuganda,org

- **Academic Alliance, Mulago, Kampala**

Professor Addy Kekitiinwa (ARROW PI) Tel: + 256 41 541 044
Department of Paediatrics Fax: +256 41 541 044
PO Box 7072 Email: akekitiinwa@pidc.idi.co.ug
Makerere University
Kampala, Uganda
Dr Philippa Musoke (ARROW co-PI) Email: PMusoke@mujhu.org
Fred Lutwana (DART Trial Co-ordinator) Email: lutwamaf2003@yahoo.com
Ruth Namulenya (DART Trial Manager) Email: ruthnalug@yahoo.co.uk
Academic Alliance
Faculty of Medicine
Makere University
P.O Box 22418
Kampala, Uganda

- **Joint Clinical Research Centre, Kampala**

Dr Francis Ssali/Dr Cissy Kityo (DART Project Leader/co-PI)
Tel: + 256 41 270 622/283
Joint Clinical Research Centre Fax: + 256 41 342 632
PO Box 10005 Email: ssalifran@yahoo.co.uk
Kampala Uganda
Dr Victor Musiime (ARROW Project Leader) Email musiimev@yahoo.co.uk
Dinah Tumukunde (DART Trial Manager) Email: dinah@jcrc.co.ug

- **University of Zimbabwe**

Dr Andrew Reid (DART co-PI) Tel: + 263 4 791 631
Department of Clinical Pharmacology Fax: + 263 4 251 017
University of Zimbabwe Email: reid@uzcrc.co.zw
Box A178 Avondale
Harare Zimbabwe
Prof Kusum Nathoo (ARROW PI) Email: knathoo@medsch.uz.ac.zw
Dr Mutsawashe Filda Dangarembizi (ARROW Project Leader)
Email: mdanga@mweb.co.zw
Ennie Chidziva (DART Trial Manager) Email: ennie@uzcrc.co.zw

- **MRC Clinical Trials Unit**

Professor Diana Gibb (DART co-PI) Tel: + 44 20 7670 4709
MRC Clinical Trials Unit Fax: + 44 20 7670 4818
222 Euston Road E-mail: di.gibb@ctu.mrc.ac.uk
London NW1 2DA, UK Emergency contact:
+44 20 7670 4700

Andy Burke (DART Trial Manager) Email: aub@ctu.mrc.ac.uk
Dr Margaret Thomason (ARROW Trial Manager) Email: mjth@ctu.mrc.ac.uk
Dr Moira Spyer (ARROW & DART Trial Manager) Email: mjs1@ctu.mrc.ac.uk

Statisticians

- **Medical Research Council Programme on AIDS**

Leigh Anne Shafer
Medical Research Council Programme on AIDS
c/o Uganda Virus Research Institute
P.O. Box 149
Entebbe, Uganda
Tel: + 256 41 320 042
Fax: + 256 41 321 137
E-mail: leighanne.shafer@mrcuganda.org

- **MRC Clinical Trials Unit**

Dr Sarah Walker
Tel: + 44 20 7670 4726
Fax: + 44 20 7670 4818
E-mail: sarah.walker@ctu.mrc.ac.uk

Helen Wilkes
Tel: + 44 20 7670 4609
Email: helen.wilkes@ctu.mrc.ac.uk

MRC Clinical Trials Unit
222 Euston Road
London NW1 2DA, UK

CONTENTS

1. SUMMARY 9
1.1 AIM AND OBJECTIVES 9
1.2 DESIGN AND POPULATIONS 9
1.3 FOLLOW-UP 9
1.4 FLOWSHEET 10
2. BACKGROUND 11
2.1 INTRODUCTION 11
2.2 DART STUDY 11
2.3 TENOFOVIR 11
2.4 ABACAVIR 12
2.5 ZIDOVUDINE, LAMIVUDINE AND NEVIRAPINE 13
2.6 OTHER DRUGS 13
2.7 RATIONALE FOR THIS STUDY 13
3. AIM AND OBJECTIVES 13
4. DESIGN 14
4.1 DESIGN OF STUDY 14
4.2 DATA COLLECTION 15
5. SELECTION OF CHILDREN 15
5.1 INCLUSION CRITERIA 15
5.2 EXCLUSION CRITERIA 15
5.3 NUMBER AND SOURCE OF CHILDREN 15
6. ASSESSMENTS AND PROCEDURES 16
6.1 ENROLMENT PROCEDURE 16
6.2 INFANT FOLLOW-UP 16
6.3 CLINICAL EXAMINATION AND MEDICAL HISTORY 16
6.4 LABORATORY TESTS 17
6.5 HIV TESTING 17
6.6 PLASMA FOR STORAGE 19
6.7 BREASTMILK FOR STORAGE 19
6.8 URINE STORAGE 19
7. ADVERSE EVENTS 19
7.1 ADVERSE EVENTS 19
7.2 SEVERITY/GRADING OF ADVERSE EVENTS 19
8. STATISTICS 19
8.1 ANALYSIS 20
9. MONITORING 20
9.1 DIRECT ACCESS TO DATA 20
9.2 CONFIDENTIALITY 21
10. ETHICS APPROVAL 21
11. FINANCE 21
12. REFERENCES 21

Appendices:

APPENDIX 1: SAMPLE INFORMATION SHEET 22
APPENDIX 2: IFU STUDY CONSENT FORM 25
APPENDIX 3: TOXICITY GRADINGS 26
APPENDIX 4: WHO STAGING 40
APPENDIX 5: DART PREGNANCY MANUAL OF OPERATIONS 46

GLOSSARY AND ABBREVIATIONS

**CRF**	Case Report Form
**HAART**	Highly Active Antiretroviral Therapy
**ART**	Antiretroviral Therapy
**PI**	Protease Inhibitor
**NRTI**	Nucleoside Reverse Transcriptase Inhibitor
**NNRTI**	Non-Nucleoside Reverse Transcriptase Inhibitor
**ZDV**	Zidovudine
**3TC**	Lamivudine
**TDF**	Tenofovir
**ABC**	Abacavir
**NVP**	Nevirapine
**EFV**	Efavirenz
**IFU**	Infant Follow Up
**MTCT**	Mother-To-Child Transmission
**MOP**	Manual Of Operations
**APR**	Antiviral Pregnancy Registry

SUBSTANTIVE CHANGES FROM DART INFANT FOLLOW UP PROTOCOL V1.1

Major

Section 1, 3 & 4.1
- Extension of the period of follow-up of infants to the end of the DART trial. This was previously to 72 weeks of age

Section 2.2
- As the DART trial was extended to 6 years, infants will now be followed to December 31st 2008 with a rollover period until September 2009

Section 1.3, 1.4, 4.1 & 6.2
- Addition of 6 monthly follow-up visits for infants after 72 weeks of age, as indicated in the new schedule flow sheet in section 1.4

Section 1.4, 4.1 & 6.5
- Changes to DNA PCR (or RNA) testing plan; the previous routine test at 24 weeks has been moved to 48 weeks. A test is now optional at weeks 12 and 24 depending on symptoms and breastfeeding history. All other tests are as previously and a new flow diagram (section 6.5) shows the test schedule

Section 4.1 & 6
- Now includes further guidelines for enrolment and collection of retrospective and prospective data

Section 4.1
- Removal of X-rays of long bones from follow up, (noting that all bone fractures are still captured as events during routine follow up)

Section 4.1, 5.1 & 6.7
- Collection and storage of breastmilk samples has been made optional for breastfeeding mothers and is no longer a requirement of the inclusion criteria

Section 5.1 & 6.1
- Removal from inclusion criteria in section 5.1 that only infants less than 1 year of age are eligible. DART infants already born and older than 1 year are now eligible and will be offered enrolment (section 6.1)

Section 6.4
- Removal of routine urine chemistries unless clinically indicated; protocol now only requires routine urine storage as indicated in section 6.8

Appendix 1
- Changes to patient information sheet template (Appendix 1) in line with protocol version 1.2

Appendix 2
- Changes to study consent form (Appendix 2) in line with protocol version 1.2 and to include breastmilk sampling and storage as optional

Appendix 3
- Toxicity gradings have been updated. Use of WHO (2007) rather than DAIDS (2004) gradings for neutrophil toxicity to recognise that infants and children of African origin have a lower normal range of absolute neutrophil counts

Appendix 4
- WHO staging has been updated

Minor

Main contacts
- These have been updated

Section 2.3-2.7
- Updates to information on research into ARV usage for prevention of mother to child transmission

1. Summary

1.1 AIM and OBJECTIVES

The overall aim of this study is to identify HIV infected pregnant women in the DART trial who are receiving triple drug ART, and to follow their infants to the end of the DART study. Infants born to women in the adult DART study will be monitored for growth and development, renal abnormalities and bone fractures, grade 3 and 4 adverse events, HIV status and survival. Any infected infants needing ART may be enrolled into the AntiRetroviral Research fOr Watoto (ARROW) trial if eligible.

The specific objectives are:

- To determine clinical, haematological, biochemical and bone adverse effects in infants born to women receiving ART in DART.

- To monitor growth and development of infants including height, weight and developmental milestones.

- To determine rates of HIV infection in infants born to women in the DART study using DNA or RNA PCR tests performed in real-time or retrospectively on stored specimens.

- To measure antiretroviral drug levels (TDF, ZDV, 3TC, ABC and NVP in particular) in breast milk in women who breastfeed and to undertake drug level measurements in plasma from their infants.

1.2 DESIGN and POPULATIONS

DART Infant Follow Up (IFU) is a longitudinal observational study. All infants born to mothers in the DART study will be approached for enrolment and followed up until completion of the DART Trial.

1.3 FOLLOW-UP

All prospectively enrolled children will be seen at around 2 weeks after birth for completion of the DART Case Record Form (CRF) for Pregnancy Outcome and enrolment into the IFU study. Enrolled infants will also be seen at weeks 6, 12 and 24 after birth, and then every 24 weeks which will coincide with the mother's scheduled DART visit. Infants already born to DART mothers before the start of IFU will be followed up prospectively according to their age and the above visits and data also collected retrospectively. The protocol scheduled visits can be seen in the flowsheet in section 1.4.

1.4 FLOWSHEET

**Evaluation**	**Mother Enrolment**	**Infant age in weeks**												
		**2**	**6**	**12**	**24**	**48**	**72**	**96**	**120**	**144**	**168**	**192**	**216**	**240**
Signed informed consent	X													
Clinical assessment [a]		X	X	X	X	X	X	X	X	X	X	X	X	X
Biochemistry I [b] *(2ml)*			X		X	X	X	X	X	X	X	X	X	X
Biochemistry II [b]			X		(X)	(X)	(X)	(X)	(X)	(X)	(X)	(X)	(X)	(X)
Haematology [c] *(1ml)*			X		X	X	X	X	X	X	X	X	X	X
Urine storage *(up to 2ml)* and dipstick test [d]			X		X	X	X	X	X	X	X	X	X	X
HIV DNA or RNA PCR [e] *(1ml)*			X	(X)	(X)	X								
HIV ELISA/rapid test							X							
Specimen storage [f] *(1ml+remainder) or blood spot if not enough blood to store*		(X)	X		X	X	X	X	X	X	X	X	X	X
Storage of breast milk samples from mother (optional)	(X)	(X)	(X)	(X)	(X)									
Cotrimoxazole[g]			X	X	X	X	(X)	(X)	(X)	(X)	(X)	(X)	(X)	(X)

If a child is seen more frequently than indicated, a prospective follow-up form should be completed at each visit.

The flow sheet indicates the ideal in terms of tests performed. At minimum, if a child enrols at or before 6 weeks of age then tests should be undertaken at 6 weeks and again at 24 and 72 weeks and then at 24 weeks following this. If a child enrols at a later age then please refer to the DART IFU Manual Of Operations (MOP, version 1.1) CRF guidelines

(a) Clinical assessment: including medical history, height, weight, head circumference, motor development, occurrence of fractures or other Grade 3 or 4 adverse events, and presence of any HIV-related signs and symptoms (Appendix 4)

(b) Biochemistry I: creatinine, BUN, AST or ALT, sodium, potassium (2ml)
Biochemistry II: where possible, calcium, phosphate, alkaline phosphatase from the same sample as Biochemistry I

(c) Haematology: haemoglobin, MCV, WBC, lymphocytes, neutrophils, platelets (1ml)

(d) Urine storage/dipstick: store 2mls of urine if possible which can be obtained from nappy/diaper if necessary. Perform dipstick test prior to storage

(e) DNA (or RNA) PCR: at 6 weeks; if positive, confirm with a second test immediately; if negative and unsure if breastfeeding or not confirm negative at 24 weeks. Repeat PCR at 48 weeks in all asymptomatic patients. Test if symptomatic at any time or if breastfeeding has ceased since the last visit. Exact time of testing babies can be flexible and take into account other factors including wish for caregivers to test earlier. See flow diagram in section 6.5

(f) Plasma storage: from a total 5ml (1 teaspoon, includes remainder from haematology/biochemistry) blood draw to be separated and stored locally at -80°C (see DART MOP for instructions for plasma handling and storage); cell pellet should be stored if feasible.

(g) Cotrimoxazole prophylaxis according to WHO guidelines (Guidelines on co-trimoxazole prophylaxis for HIV-related infections among children, adolescents and adults in resource-limited settings, WHO 2006): continue until HIV infection can be excluded by HIV antibody testing (beyond 18 months of age) or virological testing (before 18 months of age). If breastfeeding, HIV infection must be excluded at least 6 weeks after cessation of feeding.

For all tests/samples/assessments: those in brackets are optional. If possible store a specimen sample at week 2 (or use heel prick for dried blood spot storage)

2. Background

2.1 Introduction

Mother to child HIV transmission (MTCT) remains the major mode of acquisition of HIV infection in children world-wide. The vertical transmission rates in Africa tend to be much higher than the developed world. More advanced disease and almost universal breastfeeding contribute to these higher rates. In developed countries implementation of PMTCT programs is a reality; most infected pregnant women are identified early and antiretroviral therapy provided to prevent MTCT. With more effective HIV care and treatment including effective antiretroviral therapy (ART), the risk of MTCT has been reduced to less than 2% in well resourced countries (1). However, African women do not have universal access to HIV testing in pregnancy and when available, acceptance of HIV testing and use of antiretroviral drugs for PMTCT is lower, typically between 40-80%. Therefore, understanding the impact of effective ART in pregnancy in an African population is a priority.

2.2 DART study

DART (Development of Antiretroviral Therapy) is a six year clinical endpoint randomised trial to evaluate monitoring practice and structured treatment interruptions in the management of ART in adults with HIV infection in Africa (www.ctu.mrc.ac.uk/dart). Among 3315 patients (2155 women) enrolled (recruitment completed October 2004), 2468 (74%) started combivir (co-formulated zidovudine (ZDV) and lamivudine (3TC)) plus tenofovir (TDF), 547 (16%) started ZDV+3TC plus nevirapine (NVP) and 300 (9%) started ZDV+3TC plus abacavir (ABC). Pregnant women were not eligible for enrolment into DART. However, some women have become pregnant during the study follow-up period. The DART International Coordinating Group has developed a manual of operations (MOP, appendix 5) for the management of pregnancy and delivery in DART, including the reporting of pregnancies and of their outcome.

The MOP states that for women on first-line tenofovir + combivir, NVP should be given to the infant only within 4hours of birth, as it will not have been given to the mother. Babies could also receive ZDV+/-3TC for 1 week as well as this single dose of NVP, starting within 4 hours of birth. However, giving all three may be difficult as liquid formulations have to be obtained. NVP could be given as part of a national program. For mothers taking NVP+ZDV+3TC, NVP post-exposure prophylaxis should be given for the baby starting at 48 hours. ZDV+/- 3TC could be added for 1 week, if this is feasible and available, as there are few other alternative regimens. Sites may deviate from this policy if individual circumstances indicate an alternative (see MOP, appendix 5, for further details).

Although the DART MOP recommends good clinical practice for following babies born to HIV-infected women, ethics approval for DART does not cover consent for infant follow-up or collection of data on infants. Therefore it is critical to have a standard protocol to monitor the infants born to mothers in DART and to evaluate longer-term outcomes in these infants; particularly in those women receiving ZDV+3TC+TDF and ZDV+3TC+ABC where data are more limited.

2.3 Tenofovir

Tenofovir disoproxil fumarate (tenofovir DF; Viread), an ester prodrug of the nucleotide reverse transcriptase inhibitor (NRTI) tenofovir, is indicated in combination with other antiretroviral agents in the treatment of HIV infection. As a component of an antiretroviral regimen, oral tenofovir DF 300mg once daily is recommended. Tenofovir DF provides a simple and convenient once-daily dosage regimen, and is generally well tolerated and able to produce sustained suppression of viral replication.

Animal Studies of Tenofovir in Pregnancy
Reproduction studies have been performed in rats and rabbits at doses up to 14 and 19 times the human dose based on body surface area comparisons and revealed no evidence of impaired fertility or harm to the foetus due to tenofovir. There are, however, no adequate and well-controlled studies in pregnant women. Because animal reproduction studies are not always predictive of human response, TDF should be used during pregnancy only if clearly needed (Viread Prescribing Information, Gilead Sciences 19th May 2008).

Placental transfer of TDF has been studied in pregnant rhesus monkeys. Following daily subcutaneous doses of 30 mg/kg/day beginning on day 80 (beginning of second trimester) of gestation, the fetal/maternal ratio tenofovir concentrations was approximately 17% with measurements performed at gestational age 120 and 140 days (2). In addition, after subcutaneous administration of a single dose of tenofovir (30 mg/kg) to near-term pregnant macaques, the tenofovir level in the cord blood collected during C-section 2 hours later was 60% of that in maternal blood (3). In this study, infant concentrations were a mean of 5.82 +/- 1.4 mcg/ml. In addition, infant rhesus monkeys demonstrate a 5-fold decrease in clearance of tenofovir. The lower clearance is thought to be due to an immature kidney and suggests delayed excretion may also occur in neonates.

Secretion in Breast Milk
The Centers for Disease Control and Prevention recommend that HIV-infected mothers do not breast-feed their infants to avoid risking postnatal transmission of HIV. Studies in rats have demonstrated that tenofovir is secreted in milk. It is not known whether tenofovir is excreted in human milk. Because of both the potential for HIV transmission and the possibility for adverse reactions in nursing infants, Viread Prescribing Information (Gilead Sciences 19th May 2008) recommends that mothers are instructed not to breast-feed if they are receiving TDF.

Human Experience of Tenofovir
More than 12,000 patients have been treated with TDF alone or in combination with other antiretroviral medicinal products for periods of 28 days to 215 weeks in Phase 1-3 clinical trials and expanded access studies. A total of 1,544 patients have received TDF 300 mg once daily (QD) in Phase 1-3 clinical trials; over 11,000 patients have received TDF in expanded access studies (Viread U.S. Prescribing Information, Gilead Sciences 19th May 2008). Tenofovir is usually well tolerated and the most common side effects include rash, diarrhoea headache, pain, depression, asthemia and nausea. Other side effects are rare but include osteoporosis, osteopenia, renal failure, lactic acidosis, hepatomegaly with steatosis and fat redistribution.

Gilead Sciences contributes to the Antiviral Pregnancy Registry (APR) and uses this registry for tracking and following-up of all cases of pregnancy reported in patients receiving TDF in clinical practice. As of 31st July January 2006, a total of 231 post-marketing reports of pregnancies have been reported (4), with no evidence of any increase in congenital abnormalities (note that numbers are very small). Other pregnancy registers include increasing number of infants born to mothers taking Tenofovir (e.g. 75 babies in the National Study of HIV in Pregnancy and Childhood, P Tookey, personal communication). Overall in the UK study of around 4000 births, there was no evidence of an increase in congenital malformations (5). Numbers are too small to evaluate individual drugs or regimens.

2.4 Abacavir
This nucleoside reverse transcriptase inhibitor (NRTI) is also well tolerated but the hypersensitivity syndrome is one of its major side effects, reported in 3-5% of those on the drug. The hypersensitivity syndrome presents with fever, generalized rash, headache, nausea, fatigue and multi-organ involvement. Immediate and permanent discontinuation of

the drug is required and reintroduction of the drug is fatal. Therefore ABC should NEVER be reintroduced if the drug was discontinued because of the hypersensitivity syndrome.

In the Antiviral Pregnancy Registry (APR), there were outcomes reported in 436 women taking ABC during the first trimester and 697 during the second and third trimesters (4). The numbers with first-trimester exposure are sufficient to detect a 2-fold increase in congenital abnormalities compared with normal children, and no such increase was observed.

2.5 Zidovudine, Lamivudine and Nevirapine

Zidovudine and lamivudine are NRTIs that have been used for prevention of mother to child transmission with no infant adverse events related to the drug. Therefore one would not expect serious adverse events due to the maternal dosing during pregnancy and subsequent exposure through breast milk. Haemoglobin did decrease by about 1 g/dl during the 6 weeks when infants received continuous ZDV as post-exposure prophylaxis. However, this recovered as soon as ZDV was stopped in the ACTG 076 trial of ZDV to reduce MTCT (6).

Nevirapine has been widely used to reduce MTCT in late pregnancy. In the APR, over 6331 outcomes of pregnancy were reported to July 31st 2006, 2272 infants had maternal exposure to ARVs in the first trimester, 515 of these being to nevirapine. No significant increase in congenital abnormalities was observed (power to detect a 2-fold increase).

2.6 Other Drugs

Women may be on other drugs (e.g. for second line therapy) and it would be valuable to also follow infants born to these women.

2.7 Rationale for this study

Infants exposed to ART in pregnancy and during breastfeeding need to be monitored to ensure safety, particularly of TDF which is a relatively new antiretroviral drug, and is not yet licensed for use in children. As stated in the Viread Prescribing Information, TDF is placed in Pregnancy category B. The use of TDF in pregnant women has not been clearly established and studies such as the phase II trial on Tenofovir/Emtricitabine for PMTCT in Africa and Asia are small and still in recruitment stages so currently, there are no adequate and well-controlled studies of TDF in pregnant women. Therefore every effort will be made to document any adverse events that may occur during the infant follow up. In addition, documentation of the effectiveness of ART given to the mother during pregnancy and breastfeeding, for her own disease, in reducing MTCT would be of value. It is acknowledged that these data will be descriptive only, and the accuracy of the estimate of vertical HIV transmission will depend on numbers (likely to be <300 mother-infant pairs). The need to follow and monitor the growth and development of infants born to mothers taking ART in pregnancy is recognized. In particular, as babies will have been exposed to TDF during intrauterine life and during breast-feeding, the need to monitor for renal function and where possible, bone abnormalities, is also critical.

3. Aim and Objectives

The overall aim of this study is to identify HIV infected pregnant women in the DART trial who are receiving triple drug ART, and to follow their infants to the end of the DART study. Infants born to women in the adult DART study will be monitored for growth and development, renal abnormalities and bone fractures, grade 3 and 4 adverse events, HIV status and survival. Any infected infants needing ART may be enrolled into the AntiRetroviral Research fOr Watoto (ARROW) trial if eligible.

The specific objectives are:

1. To determine any clinical, haematological, biochemical and bone adverse effects in infants born to women receiving ART in DART.

2. To monitor growth and development of infants including height, weight and motor developmental milestones.

3. To determine rates of HIV infection in infants born to women in the DART study using DNA or RNA PCR tests performed in real-time or retrospectively on stored specimens.

4. To measure antiretroviral drug levels (TDF, ZDV, 3TC, ABC and NVP in particular) in breast milk in women who breastfeed and to undertake drug level measurements in plasma from their infants.

This protocol outlines a proposal for follow-up of babies born to women in the DART study. In addition (but not part of the current proposal), we plan to apply for funding for a proposal to undertake plasma pharmacokinetic studies in mothers taking TDF containing regimens, and their infants.

4. Design

4.1 Design of study

This observational study will enrol infants born to mothers in the adult DART trial. As of June 2005, 76 women had been reported to have become pregnant (incidence about 2.3 per 100 woman years at risk), but other women are likely to become pregnant before the end of the study.

All infants born to women who are pregnant or become pregnant during the DART study will be eligible for IFU study. All pregnant women in the DART study and DART mothers who have given birth to an infant before the start of IFU will be counselled about IFU including the study procedures, benefits and risks. If they agree to participate and sign the informed consent form the infant will be enrolled. At birth the delivery information, gestational age and general condition will be documented, including method of infant feeding chosen by the mother. This information will be collected retrospectively for infants already born to DART mothers prior to the start of IFU. A maternal blood sample is taken close to the time of delivery for storage in the main DART trial. At all visits the infants will have a history and physical exam. Infant blood will be collected for HIV DNA or RNA PCR during follow-up and for an HIV rapid test at 72 weeks to confirm final HIV status. If infants enrol past 72 weeks of age, then bloods will be taken for the HIV ELISA or rapid test only.

Consent will be obtained from pregnant woman prior to delivery, or if infants are already born, signed consent will be obtained from the mother/carer before the infant is enrolled in the study. Breastfeeding DART mothers may be asked for breast-milk samples but this is not a requirement for entry into IFU.

In the instances where an infant has died, the mother/carer may be approached for retrospective information. The decision to do this will be that of sites and at the clinicians discretion and will only be done if considered appropriate.

Aim 1: Monitor clinical, haematological, biochemical and bone adverse events in infants exposed to Tenofovir and/or other antiretroviral drugs in utero and through breast milk.

At each routine visit the infants will have a history and examination to document clinical adverse events and blood will be collected to document haematological and biochemical adverse events. Data will also be collected retrospectively where available.

Aim 2: Monitor growth and development of infants
Infants born to DART mothers will be seen at 2, 6, 12, and 24 weeks after delivery and then every 24 weeks until the end of the DART study. Visits should coincide with the mother's DART visit. Infants born before the start of IFU should follow the designated schedule (see flowsheet in section 1.4) according to their age. History and clinical exam including heights and weights will be documented at each routine visits and plotted on the standard child growth charts (for example, WHO). Developmental milestones will be monitored clinically – in particular motor milestones (using modified Bailey) will be documented. Study follow up visits will be done at the DART follow up clinic for all scheduled visits but the routine health care will be provided at other clinics.

Aim 3: To determine rates of HIV infection in infants
Since all the women will be receiving ART during pregnancy, the number of infants who will become infected is likely to be very low. Blood will be taken to determine the HIV status using DNA or RNA PCR (either in real-time or retrospectively) at 6 and 48 weeks If the results are positive then repeat HIV testing will be done to confirm the HIV status. Additional testing will be done at 24 weeks if negative but breastfeeding status is unknown. Infants should be tested at any time if symptomatic or if breastfeeding has stopped since the last visit. The final HIV test will be done at 72 weeks (or later if the child enrols after 72 weeks of age) using the standard HIV ELISA or HIV rapid tests. Plasma will be stored at all visits except week 12 for subsequent tests relating to HIV infection.

Aim 4: To determine the antiretroviral drug concentrations in breast milk samples
After delivery mothers in DART will be asked to express breast milk at the 2, 6, 12 and 24 week time points and at the time of stopping breast-feeding (unless close to one of the other time-points). This is optional and not a requirement for enrolling into IFU. If mothers are breastfeeding at enrolment they will also be asked for breastmilk samples, which again is optional. The breast milk samples will be frozen and stored for later assays to evaluate Tenofovir, Lamivudine, Zidovudine and Abacavir drug levels as appropriate.

4.2 Data collection

Data will be recorded CRFs, the top copy/original should be sent to the Local DART Trials Centre for data entry and a copy kept at the local clinical centre. The type of data to be recorded is detailed in the Assessments and Procedures section (Section 6).

5. Selection of Children

5.1 Inclusion criteria

i. Infants born to women in the DART study <u>OR</u> pregnant woman at term, enrolled in the DART study
ii. Mother signed the informed consent form for her infant's follow up
iii. Mother willing to comply with the follow up study procedures and visits

5.2 Exclusion criteria

None

5.3 Number and source of children

It is planned to recruit all infants born to mothers in the DART study.

6. Assessments and Procedures

6.1 Enrolment Procedure

Pregnant women from the DART study will be identified at their respective centres and consent obtained late in pregnancy to enrol their infant into this follow up study. All pregnant women, close to term, will be given information about the objectives and rationale of the study and the possible risks (see the patient information sheets in Appendix 1). Women who have already given birth when the study starts will also be able to enrol their children into the study and will be given the patient information sheets. Retrospective and prospective data is to be collected from these children.

Written informed consent to enrol into IFU will be obtained after explanation of the aims, methods, benefits and potential hazards of the study and BEFORE any study specific procedures are performed or any blood is taken for the study (see sample study consent form - Appendix 2). It will be made completely and unambiguously clear to the mother that they are free to refuse to allow their child to participate in the study, or withdraw their consent at any time and for any reason, without incurring any penalty or affecting the treatment of them or their child.

Signed informed consent forms will be kept by the investigator and documented in the case report form and a copy given to the mother.

6.2 Infant follow-up

The infant follow up visits will occur at the same site as the mother's routine DART visits (Entebbe, JCRC/Mulago, and Zimbabwe). The initial visit will occur at week 2 after delivery for pregnant women enrolled, when the DART outcome of pregnancy form will be completed (see DART pregnancy MOP, Appendix 5). Subsequent routine visits will be done at 6, 12, 24, and at 24 week intervals following this after birth, depending on the age of the infant at study entry. Follow up will continue until the completion of DART. An IFU Retrospective/Enrolment form will need to completed at the infants first visit, or, for infants born before the start of the study this may also be completed at a previous DART mothers visit. An IFU Prospective/Follow Up form should also be completed at the child's first visit and then at every subsequent visit. For full CRF guidelines see the DART IFU MOP v1.1 section 9.1 and Appendix 8

At 6 weeks of age, all infants will be started on Cotrimoxazole for PCP prophylaxis and remain on it unless their HIV status is confirmed negative following WHO guidelines: HIV infection can be excluded by HIV antibody testing (beyond 18 months of age) or virological testing (before 18 months of age), if breastfeeding, HIV infection must be excluded at least 6 weeks after cessation of feeding. The scheduled infant HIV tests can be seen in the flow diagram in section 6.5. Infants will receive treatment for acute illnesses at other clinics and only bring the infant to the DART clinic for the scheduled study visits.

The flowchart (section 1.4) indicates the scheduled protocol assessments.

6.3 Clinical examination and medical history

A clinical examination will be performed at all follow-up protocol visits. At each visit the following should be recorded:

- Body weight, height, head circumference, mid-upper arm circumference, temperature
- Motor developmental milestones (modified Bailey)
- Hospitalisations and/or Outpatient clinic attendances
- Any grade 3 or 4 adverse event since last protocol visit

- Occurrence of solicited adverse events of any grade; namely fractures, clinical anaemia
- HIV status when known
- New signs and symptoms since last protocol visit
- Hospital admissions since the last protocol visit
- Feeding method since last protocol visit
- Concomitant medication since last protocol visit

If the child has died details will be recorded.

6.4 Laboratory tests

Where blood is taken at a scheduled protocol visit, safety monitoring tests will include:

Haematology:	Haemoglobin, MCV, platelets<br>white cell count, neutrophil and lymphocyte counts
Biochemistry:	Creatinine, BUN, ALT (SGOT) or AST (SGPT), sodium, potassium; Calcium, phosphate and alkaline phosphatase will be measured if possible

(any other values provided by labs will also be recorded but may differ across sites)

6.5 HIV testing

Blood will be taken to determine the HIV status using DNA or RNA PCR at 6 weeks. RNA PCR may be used if available and easier at sites. If the infant is negative but breastfeeding, or if breastfeeding status unknown then a second PCR will be done at 24 weeks to confirm the negative test result. All infants will have a repeat PCR at 48 weeks. A PCR will also be done if an infant has signs or symptoms of HIV infection at any time or the mother has stopped breastfeeding since the last visit.

If the infant has a positive HIV DNA or RNA PCR test result, a repeat blood sample will be collected as soon as possible to confirm the HIV status of the infant. An infant will be considered HIV infected after two consecutive positive HIV DNA or RNA PCR tests: further DNA or RNA tests need not then be performed. If the infant is asymptomatic through follow up, a HIV antibody test will be done at 72 weeks using the standard HIV ELISA or HIV rapid tests. After 72 weeks further antibody tests will only be done if a child develops symptoms, is still breastfeeding (in which case a HIV antibody test will be done every 6 months) or 6 weeks after stopping breastfeeding, or if a child is enrolled at an age greater than 72 weeks and is untested. Please refer to the HIV testing flow diagram below.

Infected infants will be followed and may be eligible to join the AntiRetroviral Research fOr Watoto (ARROW) trial depending on their clinical/immunological status.

FLOW DIAGRAM OF INFANT FOLLOW UP HIV TESTING

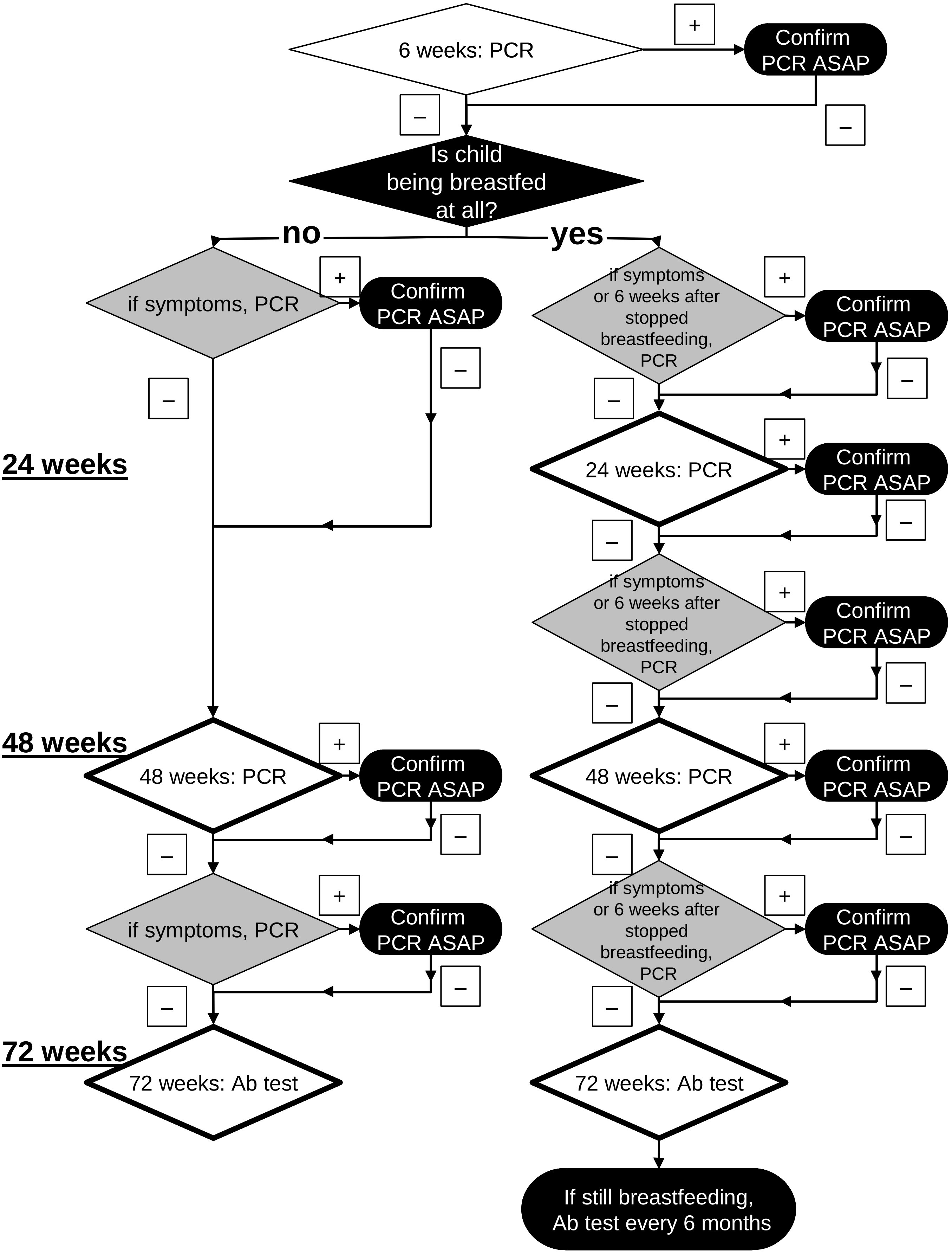

6.6 Plasma for storage

1ml of EDTA blood will be collected at each protocol visit for plasma storage for retrospective analysis of HIV or any treatment related tests as appropriate (for example, HIV resistance testing or antiretroviral drug levels). A cell pellet will also be stored if possible. This blood will be the remainder of a total 5ml blood draw for storage and other tests.

6.7 Breastmilk for storage

The collection of breastmilk from breast-feeding women will be optional. If agreed, approximately 15 mls of breast milk will be expressed from each breast at 2, 6, 12 and 24 weeks after delivery (depending on when breast feeding is stopped). The breast milk will be frozen and stored for later testing of antiretroviral drug levels.

6.8 Urine storage

Up to 2mls of urine will be collected from infants at weeks 6, 24 and then every scheduled 24 week visit. This will be frozen and stored for retrospective analysis.

7. Adverse events

7.1 Adverse events

Adverse events (AEs) are any untoward medical occurrences whether or not they are necessarily caused by or related to a medicinal product. Adverse events may be expected or unexpected. They may include side-effects, injury, toxicity or sensitivity reactions, abnormal laboratory results and intercurrent illnesses. An adverse reaction is an adverse event which is related to any dose administered to that subject. An unexpected adverse reaction is an adverse reaction, the nature and severity of which is not consistent with the information about the medicinal product in question set out in the SPC for that product (for products with a marketing authorisation) or the Investigator's Brochure relating to the study in question (for any other investigational product).

It is particularly important to monitor adverse events closely in infants born to mothers in the DART study, as there is relatively little information about two of the drugs (TDF and ABC) that some pregnant women will have received. Therefore the follow-up form will explicitly solicit information about certain adverse events; namely fractures and clinical anaemia.

7.2 Severity/grading of adverse events

Standard criteria for grading laboratory and clinical events will be used for grading adverse events (Appendix 3). Adverse events will be categorised as HIV related, drug related and others. Any adverse events of grade 3 or 4 will be reported on the IFU Retrospective/Enrolment and Prospective Follow-Up form.

8. Statistics

Because this study is observational, no analysis was conducted to determine the sample size requirement to test a hypothesis. Instead, we show below the precision with which such a study could estimate HIV incidence and adverse events.

There are 2155 women enrolled in the DART study: if the current pregnancy rate of 2.3 per 100 woman years continues, then we would expect to see 150 pregnancies by the end of the study. However, there were virtually no pregnancies in the first year of the study, and anecdotally more women are enquiring about becoming pregnant. We therefore estimate

that there might be 200-400 pregnancies in women in the DART study. However, a number of pregnancies so far have not been carried to term. Therefore we expect anywhere between 100-400 infants born to women in the DART study.

The following table shows the precision (95% C.I.) that one would obtain for a wide range of HIV transmission rates if 100-400 infants were enrolled in the study. Exact tests were used to determine the 95% confidence intervals.

	**100 infants**	**200 infants**	**300 infants**	**400 infants**
**Transmission**	**95% C.I.**	**95% C.I.**	**95% C.I.**	**95% C.I.**
**1%**	(0.0%, 5.4%)	(0.1%, 3.6%)	(0.2%, 2.9%)	(0.3%, 2.5%)
**2%**	(0.2%, 7.0%)	(0.5%, 5.0%)	(0.7%, 4.3%)	(0.9%, 3.9%)
**4%**	(1.1%, 9.9%)	(1.7%, 7.7%)	(2.1%, 6.9%)	(2.3%, 6.4%)
**6%**	(2.2%, 12.6%)	(3.1%, 10.2%)	(3.6%, 9.3%)	(3.9%, 8.8%)
**8%**	(3.5%, 15.2%)	(4.6%, 12.7%)	(5.2%, 11.7%)	(5.5%, 11.1%)

The following table shows the precision (95% C.I.) that one would obtain for a wide range of adverse event rates if 100-400 infants were enrolled in the study. Exact tests were used to determine the 95% confidence intervals.

	**100 infants**	**200 infants**	**300 infants**	**400 infants**
**AE rate**	**95% C.I.**	**95% C.I.**	**95% C.I.**	**95% C.I.**
**10%**	(4.9%, 17.6%)	(6.2%, 15.0%)	(6.8%, 14.0%)	(7.2%, 13.4%)
**15%**	(8.6%, 23.5%)	(10.4%, 20.7%)	(11.2%, 19.6%)	(11.6%, 18.9%)
**20%**	(12.7%, 29.2%)	(14.6%, 26.2%)	(15.6%, 25.0%)	(16.2%, 24.3%)
**25%**	(17.9%, 34.7%)	(19.2%, 31.6%)	(20.2%, 30.3%)	(20.8%, 29.5%)
**30%**	(21.2%, 40.0%)	(23.7%, 36.8%)	(24.9%, 35.5%)	(25.5%, 34.8%)
**35%**	(25.7%, 45.2%)	(28.4%, 42.0%)	(29.6%, 40.7%)	(30.3%, 39.9%)

8.1 Analysis

Data will be entered at each clinical site, and merged centrally at the MRC CTU. Statistical analysis of results will be undertaken under the supervision of senior statisticians at MRC CTU in collaboration with paediatricians and DART trial team members from Mulago Hospital, Entebbe, Harare and JCRC.

Rates and cumulative proportions of HIV transmission, adverse events and death will be described using standard time to event methods. Longitudinal changes in growth and laboratory parameters will be described, and comparisons made between the HIV infected and serorevertors (uninfected) from Zimbabwe and Uganda. Analysis of growth parameters will be adjusted for age (z-scores).

It is not expected that comparisons between infected and uninfected children will be possible due to low transmission rates.

9. Monitoring

9.1 Direct Access to Data

Personal medical data may be reviewed at clinical centres by properly authorised individuals as part of monitoring and/or audit of the study, but such information will be treated as strictly confidential and will in no circumstances be made publicly available.

9.2 Confidentiality

The child's anonymity will be maintained. On all CRFs and specimens, children must not be identified by their names. Children will be identified by a unique substudy number, their mother's DART Trial number, their initials and date of birth. The investigator will be asked to keep a separate Confidential Register which matches these identifiers with their names and which should be maintained by the investigator in strict confidence.

10. Ethics Approval

At each centre, one DART site investigator and one paediatrician will take on overall responsibility for the conduct of the study. They will submit the protocol and any subsequent amendments to the ethics committee.

This protocol and the informed consent documents will be formally approved by the relevant ethics committee of each clinical centre and by a national ethics body, as required. A copy of the written approval must be sent to the MRC Clinical Trials Unit before the study starts in that centre.

11. Finance

Research support will be provided to the clinical centres for additional visits and sample collection.

12. References

1. The European Collaborative Study. Mother-to-child transmission of HIV infection in the era of highly active antiretroviral therapy Clin Infect Dis. 2005 Feb 1;40(3):458-65.

2. Tarantal, A. F., M. L. Marthas, et al. Administration of 9-[2-(R)-(phosphonomethoxy)propyl]adenine (PMPA) to gravid and infant rhesus macaques (Macaca mulatta): safety and efficacy studies. JAIDS 1999; 20:323-333.

3. Van Rompay KKA, Marthas ML, Lifson JD et al. Administration of 9-[2-(phosphhonomethoxy)propyl] adenine (PMPA) for prevention of perinatal simian immunodeficiency virus infection in phesus macaques. AIDS Res Hum Retroviruses 1998; 14:761-773.

4. The Antiretroviral Registry interim report 1 Jan 1989 through 31st July 2006 (issued Dec 2006).

5. Tookey PA, Townsend CL, Cortina-Borja M, Masters JE, Peckham CS. Antiretroviral therapy and pregnancy outcome: UK/Ireland surveillance data 1990-2004. Int Cong Drug Therapy HIV 2004 Nov 14-18; Abstract No. PL11.3.

6. Sperling RS, Shapiro DE, McSherry GD, Britto P, Cunningham BE, Culnane M, et al. Safety of the maternal-infant zidovudine regimen utilized in the Pediatric AIDS Clinical Trial Group 076 Study. AIDS 1998; 12(14):1805-13.

Appendix 1: Sample information sheet

(version 1.2)

(to be printed on headed paper and translated as necessary)

Follow up of infants born to HIV infected women in the DART study

An information sheet for mothers/carers

"Helping you decide whether or not your baby should join our study"

Introduction

You and your baby are being invited to take part in this research study, which is related to the DART trial. We would like you to know about the study before you make a decision. Please read this information carefully or have someone read it to you. The study staff will talk with you about this information. You are free to ask the clinic nurses or counsellors if you would like more information or clarification. You are free to ask questions about this study at any time.

This sheet gives you information about this study. If you agree to allow your baby to take part in this study, you will be asked to sign a consent form. You will get a copy to keep.

What is the reason for this study?

During the pregnancy, the baby was exposed to antiretroviral therapy (ART). Until now there have been no reported increases in any kind of birth abnormalities in babies born to women taking ART in pregnancy. However this is still something that is being closely looked at. Some drugs have been used a lot in pregnant women and are probably safe, although the long-term effects on the baby are not yet known.

**Combivir+Nevirapine**:	Many women have become pregnant around the world whilst taking this combination: Most have remained very well, but some women and babies do get anaemic (low red cells) with ZDV.
**Combivir+Abacavir:**	There is less experience of abacavir use in pregnancy, so far no specific problems have been reported.
**Combivir+Tenofovir**:	There is less experience of TDF in pregnancy. So far no specific problems have been reported in babies. Concern that TDF could affect the baby's bones has been expressed because it has been seen in some baby animals whose mothers received TDF.

Long-term follow-up is recommended for a baby whose mother takes **anti-HIV** drugs during pregnancy.

This study will follow babies born to HIV-infected mothers taking drugs in DART, to make sure that they remain well until the end of the DART study and to check for any side effects. It is extremely rare for drugs taken in pregnancy to affect babies, but we would like to monitor this. We would also see how many babies born to mothers taking combination anti-HIV treatment actually become infected with HIV. In addition, we would like, if breastmilk is made available to us, to measure levels of anti-HIV drugs in breast milk during breast-feeding to see if this could be helping to stop babies getting infected.

What does my baby have to do in this study?

This is an observational study. This study will not provide care related to your pregnancy or the delivery of your baby. Cotrimoxazole will be prescribed to your baby from 6 weeks of age to prevent your baby getting a common chest infection (called PCP). No other treatment or clinical care will be provided in this study. If your baby gets ill, you will need to take them to your usual clinic for treatment. Any infected babies who need anti-HIV drugs may be enrolled into the ARROW study if they are eligible.

You will be asked to bring your baby to the clinic at specific visits during this study up until the end of the main DART study. Information will be collected at every visit using the prospective follow-up form and will include the following: history of development, length, weight, head size, any hospitalisations, HIV status when known, information on infant feeding and any other medication your baby has been taking. Retrospective information will also be collected at the enrolment visit.

Blood will be taken to check whether or not your baby has got HIV at 6 weeks and 48 weeks and at other times if you are still breast feeding. If your baby is well, HIV antibody testing will be done at 72 weeks. If your baby becomes sick, a blood sample will be taken at that visit to check the HIV status. If he/ she has a positive HIV test result, a repeat blood sample will be collected at the next visit to confirm that your baby has HIV. An infant will be considered HIV infected after 2 positive HIV PCR DNA or RNA tests. **You will receive the results of your baby's tests as soon as they become available.**

At week 6, blood tests will be done, and then repeated at week 24, and the other follow up visits until the end of the study, to check how well your baby's kidneys and liver are working as well as the number of red and white cells. **You will receive the results of your baby's tests as soon as they become available.** Any leftover blood will be stored at all the blood draw time points for later retrospective HIV testing and resistance testing if required.

At these visits we will take about 5mls of blood in total (approximately 1 teaspoon) from your baby. We will use a cream to make sure that their skin is numb to diminish pain.

We would also like to collect breast milk from breast-feeding women. This is optional, and if agreed then approximately 15 mls of breast milk will be expressed from each breast at 2, 6, 12 and 24 weeks after delivery (depending on when breast feeding has stopped). For breastfeeding mothers who enrol after their baby is born they may be asked for an optional sample at the enrolment visit. The breast milk will be frozen and stored for later antiretroviral drug levels measurements.

Your baby should be brought for the study visits at the following ages:

Visit week	Age of baby
2	2 weeks
6	6 weeks
12	12 weeks
24	24 weeks
48	48 weeks
72	1 year, 20 weeks
96	1 year, 44 weeks
120	2 years, 16 weeks
144	2 years, 40 weeks
168	3 years, 12 weeks
192	3 years, 36 weeks
216	4 years, 8 weeks
240	4 years, 32 weeks

These visits are aimed to coincide with the mother's visit to the DART clinic. Because the visits are at 24 week intervals some infants may not have been seen for up to 6months near the end of the DART study so we would also like to get an additional last follow up visit near to the end of the DART study. These will also coincide with the mothers/carers DART visit schedule.

Confidentiality
Information about your baby will be kept confidential and will not be made available to anyone who is not connected to the study without your consent.

Participation
Participation in this study is entirely voluntary. If you decide that you do not wish your baby to take part that is entirely your right. Your decision will in no way affect any present or future treatment for you.

What are the possible benefits and disadvantages of taking part?
Possible disadvantages are that you will have to bring your baby to the DART clinic with you. Your baby will also have to have a small amount of blood drawn (approximately 1 teaspoon) each time. It is hoped that by following up infants born in DART long term we will be able to show that taking these antiretroviral drugs during pregnancy is safe.

As part of this study, blood samples will be stored. These will be tested at the end of the study to find out how much of your drugs there are in your baby. Some tests may also look for HIV antibodies in your baby, but new tests are being developed all the time. We are therefore asking for you to agree that stored blood samples can be used in future for tests relevant to HIV.

If you need more information about this study, please ask the doctors, nurses or counsellors or call:

Doctor: .... Tel: ....

Nurse: .... Tel: ....

Counsellor: .... Tel: ....

Thank you for taking time to consider this study for your child. Please ask any questions and let us know if there are things that you do not understand, or would like more information about.

Appendix 2: IFU Study Consent Form (Version 1.2)

To be printed on headed paper

FOLLOW UP OF INFANTS BORN IN DART STUDY CONSENT

Centre: Entebbe ❍ Harare ❍ JCRC ❍ Acad All ❍

Child's Initials				Date/Year of Birth	D	D	M	M	M	2	0	Y	Y	Child's Clinic / Hospital Number								

Date of Form	D	D	M	M	M	2	0	Y	Y	Male ❍<br>Female ❍	Mother's DART Number							

DART INFANT FOLLOW UP CONSENT for PARENTS/CARER

Please initial (or mark box) if you agree:

**I have read and understood the information sheet (version 1.2) on the DART infant follow-up study. The details of this study have been explained to me by Dr………………………. and my questions have been answered satisfactorily.**	
**I know that I can withdraw my child from the study at any time.**	
**I am willing to allow access to my child's medical notes to check that the DART Infant Follow Up study is being carried out correctly and I understand that strict confidentiality will be maintained.**	
**I agree to my child's routine blood results and clinical information being included in continued follow-up after the study has ended. I understand that this information will not be identified by either my or my child's name.**	
**I agree that my child can take part in this study, which is following children whose mothers have taken combination anti-HIV therapy during pregnancy.**	
**I agree for small quantities of blood to be stored so that further work can be done to help understand more about effective prevention of HIV. I understand that these samples will not be identified by either my or my child's name.**	
Optional:	
If the child's mother and breastfeeding, I agree to provide breastmilk samples to look at levels of anti-HIV drugs which my baby might be receiving but understand that this is optional and not a requirement for my child to enter the study.	

Carer's signature (or thumbprint)	Print name	Date
		D D M M M 2 0 Y Y

Witness's signature (or thumbprint)	Print name	Date
		D D M M M 2 0 Y Y

Doctor's signature	Print name	Date
		D D M M M 2 0 Y Y

IMPORTANT: one signed original to be kept in DART Infant Follow-Up trial file by researcher, one signed copy to be given to carer, one signed original to be kept in the clinic notes

Appendix 3: Toxicity Gradings

Division of AIDS table for grading the severity of adverse events (published December 2004) apart from neutrophil gradings, which are based on WHO guidelines (Antiretroviral therapy for HIV infection in infants and children towards universal access: Recommendations for a public health approach (2007 Revision). *World Health Organization* 2007) and NIH Paediatric toxicity tables (NIH Division of Microbiology and Infectious Diseases (DMID) Pediatric toxicity tables, November 2007).

ULN = Upper Limit of Normal ; LLN = Lower Limit of Normal

CLINICAL				
**PARAMETER**	**GRADE 1<br>MILD**	**GRADE 2<br>MODERATE**	**GRADE 3<br>SEVERE**	**GRADE 4<br>POTENTIALLY LIFE-THREATENING**
**ESTIMATING SEVERITY GRADE**				
Clinical adverse event NOT identified elsewhere in this DAIDS AE grading table	Symptoms causing no or minimal interference with usual social & functional activities	Symptoms causing greater than minimal interference with usual social & functional activities	Symptoms causing inability to perform usual social & functional activities	Symptoms causing inability to perform basic self-care functions OR Medical or operative intervention indicated to prevent permanent impairment, persistent disability, or death
**SYSTEMIC**				
Acute systemic allergic reaction	Localized urticaria (wheals) with no medical intervention indicated	Localized urticaria with medical intervention indicated OR Mild angioedema with no medical intervention indicated	Generalized urticaria OR Angioedema with medical intervention indicated OR Symptomatic mild bronchospasm	Acute anaphylaxis OR Life-threatening bronchospasm OR laryngeal edema
Chills	Symptoms causing no or minimal interference with usual social & functional activities	Symptoms causing greater than minimal interference with usual social & functional activities	Symptoms causing inability to perform usual social & functional activities	NA
Fatigue<br>Malaise	Symptoms causing no or minimal interference with usual social & functional activities	Symptoms causing greater than minimal interference with usual social & functional activities	Symptoms causing inability to perform usual social & functional activities	Incapacitating fatigue/ malaise symptoms causing inability to perform basic self-care functions
Fever (nonaxillary)	37.7 – 38.6°C	38.7 – 39.3°C	39.4 – 40.5°C	> 40.5°C
Pain (indicate body site)<br>DO NOT use for pain due to injection (See Injection Site Reactions: Injection site pain)<br>See also Headache, Arthralgia, and Myalgia	Pain causing no or minimal interference with usual social & functional activities	Pain causing greater than minimal interference with usual social & functional activities	Pain causing inability to perform usual social & functional activities	Disabling pain causing inability to perform basic self-care functions OR Hospitalization (other than emergency room visit) indicated
Unintentional weight loss	NA	5 – 9% loss in body weight from baseline	10 – 19% loss in body weight from baseline	≥ 20% loss in body weight from baseline OR Aggressive intervention indicated [e.g., tube feeding or total parenteral nutrition (TPN)]

CLINICAL				
**PARAMETER**	**GRADE 1 MILD**	**GRADE 2 MODERATE**	**GRADE 3 SEVERE**	**GRADE 4 POTENTIALLY LIFE-THREATENING**
**INFECTION**				
Infection (any other than HIV infection)	Localized, no systemic antimicrobial treatment indicated AND Symptoms causing no or minimal interference with usual social & functional activities	Systemic antimicrobial treatment indicated OR Symptoms causing greater than minimal interference with usual social & functional activities	Systemic antimicrobial treatment indicated AND Symptoms causing inability to perform usual social & functional activities OR Operative intervention (other than simple incision and drainage) indicated	Life-threatening consequences (e.g., septic shock)
**INJECTION SITE REACTIONS**				
Injection site pain (pain without touching)<br>Or<br>Tenderness (pain when area is touched)	Pain/tenderness causing no or minimal limitation of use of limb	Pain/tenderness limiting use of limb OR Pain/tenderness causing greater than minimal interference with usual social & functional activities	Pain/tenderness causing inability to perform usual social & functional activities	Pain/tenderness causing inability to perform basic self-care function OR Hospitalization (other than emergency room visit) indicated for management of pain/tenderness
Injection site reaction (localized)				
**Adult > 15 years**	Erythema OR Induration of 5x5 cm – 9x9 cm (or 25 cm^2 – 81cm^2)	Erythema OR Induration OR Edema > 9 cm any diameter (or > 81 cm^2)	Ulceration OR Secondary infection OR Phlebitis OR Sterile abscess OR Drainage	Necrosis (involving dermis and deeper tissue)
**Paediatric ≤ 15 years**	Erythema OR Induration OR Edema present but ≤ 2.5 cm diameter	Erythema OR Induration OR Edema > 2.5 cm diameter but < 50% surface area of the extremity segment (e.g., upper arm/thigh)	Erythema OR Induration OR Edema involving ≥ 50% surface area of the extremity segment (e.g., upper arm/thigh) OR Ulceration OR Secondary infection OR Phlebitis OR Sterile abscess OR Drainage	Necrosis (involving dermis and deeper tissue)
Pruritis associated with injection<br>See also Skin: Pruritis (itching - no skin lesions)	Itching localized to injection site AND Relieved spontaneously or with < 48 hours treatment	Itching beyond the injection site but not generalized OR Itching localized to injection site requiring ≥ 48 hours treatment	Generalized itching causing inability to perform usual social & functional activities	NA
**SKIN – DERMATOLOGICAL**				
Alopecia	Thinning detectable by study participant (or by caregiver for young children and disabled adults)	Thinning or patchy hair loss detectable by health care provider	Complete hair loss	NA
Cutaneous reaction – rash	Localized macular rash	Diffuse macular, maculopapular, or morbilliform rash OR Target lesions	Diffuse macular, maculopapular, or morbilliform rash with vesicles or limited number of bullae OR Superficial ulcerations of mucous membrane limited to one site	Extensive or generalized bullous lesions OR Stevens-Johnson syndrome OR Ulceration of mucous membrane involving two or more distinct mucosal sites OR Toxic epidermal necrolysis (TEN)

CLINICAL				
**PARAMETER**	**GRADE 1 MILD**	**GRADE 2 MODERATE**	**GRADE 3 SEVERE**	**GRADE 4 POTENTIALLY LIFE-THREATENING**
Hyperpigmentation	Slight or localized	Marked or generalized	NA	NA
Hypopigmentation	Slight or localized	Marked or generalized	NA	NA
Pruritis (itching – no skin lesions)<br>(See also Injection Site Reactions: Pruritis associated with injection)	Itching causing no or minimal interference with usual social & functional activities	Itching causing greater than minimal interference with usual social & functional activities	Itching causing inability to perform usual social & functional activities	NA
**CARDIOVASCULAR**				
Cardiac arrhythmia (general)<br>(By ECG or physical exam)	Asymptomatic AND No intervention indicated	Asymptomatic AND Non-urgent medical intervention indicated	Symptomatic, non-life-threatening AND Non-urgent medical intervention indicated	Life-threatening arrhythmia OR Urgent intervention indicated
Cardiac-ischemia/infarction	NA	NA	Symptomatic ischemia (stable angina) OR Testing consistent with ischemia	Unstable angina OR Acute myocardial infarction
Hemorrhage (significant acute blood loss)	NA	Symptomatic AND No transfusion indicated	Symptomatic AND Transfusion of ≤ 2 units packed RBCs (for children ≤ 10 cc/kg) indicated	Life-threatening hypotension OR Transfusion of > 2 units packed RBCs (for children > 10 cc/kg) indicated
Hypertension				
**Adult > 17 years**<br>(with repeat testing at same visit)	> 140 – 159 mmHg systolic<br>OR<br>> 90 – 99 mmHg diastolic	> 160 – 179 mmHg systolic<br>OR<br>> 100 – 109 mmHg diastolic	> 180 mmHg systolic<br>OR<br>> 110 mmHg diastolic	Life-threatening consequences (e.g., malignant hypertension) OR Hospitalization indicated (other than emergency room visit)
**Paediatric ≤ 17 years**<br>(with repeat testing at same visit)	NA	91st – 94th percentile adjusted for age, height, and gender (systolic and/or diastolic)	≥ 95th percentile adjusted for age, height, and gender (systolic and/or diastolic)	Life-threatening consequences (e.g., malignant hypertension) OR Hospitalization indicated (other than emergency room visit)
Hypotension	NA	Symptomatic, corrected with oral fluid replacement	Symptomatic, IV fluids indicated	Shock requiring use of vasopressors or mechanical assistance to maintain blood pressure
Pericardial effusion	Asymptomatic, small effusion requiring no intervention	Asymptomatic, moderate or larger effusion requiring no intervention	Effusion with non-life threatening physiologic consequences OR Effusion with non-urgent intervention indicated	Life-threatening consequences (e.g., tamponade) OR Urgent intervention indicated
Prolonged PR interval				
**Adult > 16 years**	PR interval<br>0.21 – 0.25 sec	PR interval<br>> 0.25 sec	Type II 2nd degree AV block OR Ventricular pause > 3.0 sec	Complete AV block

CLINICAL				
**PARAMETER**	**GRADE 1 MILD**	**GRADE 2 MODERATE**	**GRADE 3 SEVERE**	**GRADE 4 POTENTIALLY LIFE-THREATENING**
**Paediatric ≤ 16 years**	1st degree AV block (PR > normal for age and rate)	Type I 2nd degree AV block	Type II 2nd degree AV block	Complete AV block
Prolonged QTc				
**Adult > 16 years**	Asymptomatic, QTc interval 0.45 – 0.47 sec OR Increase interval < 0.03 sec above baseline	Asymptomatic, QTc interval 0.48 – 0.49 sec OR Increase in interval 0.03 – 0.05 sec above baseline	Asymptomatic, QTc interval ≥ 0.50 sec OR Increase in interval ≥ 0.06 sec above baseline	Life-threatening consequences, e.g. Torsade de pointes or other associated serious ventricular dysrhythmia
**Paediatric ≤ 16 years**	Asymptomatic, QTc interval 0.450 – 0.464 sec	Asymptomatic, QTc interval 0.465 – 0.479 sec	Asymptomatic, QTc interval ≥ 0.480 sec	Life-threatening consequences, e.g. Torsade de pointes or other associated serious ventricular dysrhythmia
Thrombosis/embolism	NA	Deep vein thrombosis AND No intervention indicated (e.g., anticoagulation, lysis filter, invasive procedure)	Deep vein thrombosis AND Intervention indicated (e.g., anticoagulation, lysis filter, invasive procedure)	Embolic event (e.g., pulmonary embolism, life-threatening thrombus)
Vasovagal episode (associated with a procedure of any kind)	Present without loss of consciousness	Present with transient loss of consciousness	NA	NA
Ventricular dysfunction (congestive heart failure)	NA	Asymptomatic diagnostic finding AND intervention indicated	New onset with symptoms OR Worsening symptomatic congestive heart failure	Life-threatening congestive heart failure
**GASTROINTESTINAL**				
Anorexia	Loss of appetite without decreased oral intake	Loss of appetite associated with decreased oral intake without significant weight loss	Loss of appetite associated with significant weight loss	Life-threatening consequences OR Aggressive intervention indicated [e.g., tube feeding or total parenteral nutrition (TPN)]
Ascites	Asymptomatic	Symptomatic AND Intervention indicated (e.g., diuretics or therapeutic paracentesis)	Symptomatic despite intervention	Life-threatening consequences
Cholecystitis	NA	Symptomatic AND Medical intervention indicated	Radiologic, endoscopic, or operative intervention indicated	Life-threatening consequences (e.g., sepsis or perforation)
Constipation	NA	Persistent constipation requiring regular use of dietary modifications, laxatives, or enemas	Obstipation with manual evacuation indicated	Life-threatening consequences (e.g., obstruction)
Diarrhea				
**Adult and Paediatric ≥ 1 year**	Transient or intermittent episodes of unformed stools OR Increase of ≤ 3 stools over baseline per 24-hour period	Persistent episodes of unformed to watery stools OR Increase of 4 – 6 stools over baseline per 24-hour period	Bloody diarrhea OR Increase of ≥ 7 stools per 24-hour period OR IV fluid replacement indicated	Life-threatening consequences (e.g., hypotensive shock)

CLINICAL				
**PARAMETER**	**GRADE 1 MILD**	**GRADE 2 MODERATE**	**GRADE 3 SEVERE**	**GRADE 4 POTENTIALLY LIFE-THREATENING**
**Paediatric < 1 year**	Liquid stools (more unformed than usual) but usual number of stools	Liquid stools with increased number of stools OR Mild dehydration	Liquid stools with moderate dehydration	Liquid stools resulting in severe dehydration with aggressive rehydration indicated OR Hypotensive shock
Dysphagia-Odynophagia	Symptomatic but able to eat usual diet	Symptoms causing altered dietary intake without medical intervention indicated	Symptoms causing severely altered dietary intake with medical intervention indicated	Life-threatening reduction in oral intake
Mucositis/stomatitis (clinical exam)<br>Indicate site (e.g., larynx, oral)<br>See Genitourinary for Vulvovaginitis<br>See also Dysphagia-Odynophagia and Proctitis	Erythema of the mucosa	Patchy pseudomembranes or ulcerations	Confluent pseudomembranes or ulcerations OR Mucosal bleeding with minor trauma	Tissue necrosis OR Diffuse spontaneous mucosal bleeding OR Life-threatening consequences (e.g., aspiration, choking)
Nausea	Transient (< 24 hours) or intermittent nausea with no or minimal interference with oral intake	Persistent nausea resulting in decreased oral intake for 24 – 48 hours	Persistent nausea resulting in minimal oral intake for > 48 hours OR Aggressive rehydration indicated (e.g., IV fluids)	Life-threatening consequences (e.g., hypotensive shock)
Pancreatitis	NA	Symptomatic AND Hospitalization not indicated (other than emergency room visit)	Symptomatic AND Hospitalization indicated (other than emergency room visit)	Life-threatening consequences (e.g., circulatory failure, hemorrhage, sepsis)
Proctitis (functional-symptomatic)<br>Also see Mucositis/stomatitis for clinical exam	Rectal discomfort AND No intervention indicated	Symptoms causing greater than minimal interference with usual social & functional activities OR Medical intervention indicated	Symptoms causing inability to perform usual social & functional activities OR Operative intervention indicated	Life-threatening consequences (e.g., perforation)
Vomiting	Transient or intermittent vomiting with no or minimal interference with oral intake	Frequent episodes of vomiting with no or mild dehydration	Persistent vomiting resulting in orthostatic hypotension OR Aggressive rehydration indicated (e.g., IV fluids)	Life-threatening consequences (e.g., hypotensive shock)
**NEUROLOGIC**				
Alteration in personality-behavior or in mood (e.g., agitation, anxiety, depression, mania, psychosis)	Alteration causing no or minimal interference with usual social & functional activities	Alteration causing greater than minimal interference with usual social & functional activities	Alteration causing inability to perform usual social & functional activities	Behavior potentially harmful to self or others (e.g., suicidal and homicidal ideation or attempt, acute psychosis) OR Causing inability to perform basic self-care functions

CLINICAL				
**PARAMETER**	**GRADE 1<br>MILD**	**GRADE 2<br>MODERATE**	**GRADE 3<br>SEVERE**	**GRADE 4<br>POTENTIALLY LIFE-THREATENING**
Altered Mental Status<br>For Dementia, see Cognitive and behavioral/attentional disturbance (including dementia and attention deficit disorder)	Changes causing no or minimal interference with usual social & functional activities	Mild lethargy or somnolence causing greater than minimal interference with usual social & functional activities	Confusion, memory impairment, lethargy, or somnolence causing inability to perform usual social & functional activities	Delirium OR obtundation, OR coma
Ataxia	Asymptomatic ataxia detectable on exam OR Minimal ataxia causing no or minimal interference with usual social & functional activities	Symptomatic ataxia causing greater than minimal interference with usual social & functional activities	Symptomatic ataxia causing inability to perform usual social & functional activities	Disabling ataxia causing inability to perform basic self-care functions
Cognitive and behavioral/attentional disturbance (including dementia and attention deficit disorder)	Disability causing no or minimal interference with usual social & functional activities OR Specialized resources not indicated	Disability causing greater than minimal interference with usual social & functional activities OR Specialized resources on part-time basis indicated	Disability causing inability to perform usual social & functional activities OR Specialized resources on a full-time basis indicated	Disability causing inability to perform basic self-care functions OR Institutionalization indicated
CNS ischemia (acute)	NA	NA	Transient ischemic attack	Cerebral vascular accident (CVA, stroke) with neurological deficit
Developmental delay – **Paediatric ≤ 16 years**	Mild developmental delay, either motor or cognitive, as determined by comparison with a developmental screening tool appropriate for the setting	Moderate developmental delay, either motor or cognitive, as determined by comparison with a developmental screening tool appropriate for the setting	Severe developmental delay, either motor or cognitive, as determined by comparison with a developmental screening tool appropriate for the setting	Developmental regression, either motor or cognitive, as determined by comparison with a developmental screening tool appropriate for the setting
Headache	Symptoms causing no or minimal interference with usual social & functional activities	Symptoms causing greater than minimal interference with usual social & functional activities	Symptoms causing inability to perform usual social & functional activities	Symptoms causing inability to perform basic self-care functions OR Hospitalization indicated (other than emergency room visit) OR Headache with significant impairment of alertness or other neurologic function
Insomnia	NA	Difficulty sleeping causing greater than minimal interference with usual social & functional activities	Difficulty sleeping causing inability to perform usual social & functional activities	Disabling insomnia causing inability to perform basic self-care functions
Neuromuscular weakness (including myopathy & neuropathy)	Asymptomatic with decreased strength on exam OR Minimal muscle weakness causing no or minimal interference with usual social & functional activities	Muscle weakness causing greater than minimal interference with usual social & functional activities	Muscle weakness causing inability to perform usual social & functional activities	Disabling muscle weakness causing inability to perform basic self-care functions OR Respiratory muscle weakness impairing ventilation

CLINICAL				
**PARAMETER**	**GRADE 1 MILD**	**GRADE 2 MODERATE**	**GRADE 3 SEVERE**	**GRADE 4 POTENTIALLY LIFE-THREATENING**
Neurosensory alteration (including paresthesia and painful neuropathy)	Asymptomatic with sensory alteration on exam or minimal paresthesia causing no or minimal interference with usual social & functional activities	Sensory alteration or paresthesia causing greater than minimal interference with usual social & functional activities	Sensory alteration or paresthesia causing inability to perform usual social & functional activities	Disabling sensory alteration or paresthesia causing inability to perform basic self-care functions
Seizure: (new onset) – **Adult ≥ 18 years**<br>See also Seizure: (known pre-existing seizure disorder)	NA	1 seizure	2 – 4 seizures	Seizures of any kind which are prolonged, repetitive (e.g., status epilepticus), or difficult to control (e.g., refractory epilepsy)
Seizure: (known pre-existing seizure disorder) **– Adult ≥ 18 years**<br>For worsening of existing epilepsy the grades should be based on an increase from previous level of control to any of these levels.	NA	Increased frequency of pre-existing seizures (non-repetitive) without change in seizure character OR Infrequent break-through seizures while on stable medication in a previously controlled seizure disorder	Change in seizure character from baseline either in duration or quality (e.g., severity or focality)	Seizures of any kind which are prolonged, repetitive (e.g., status epilepticus), or difficult to control (e.g., refractory epilepsy)
Seizure **– Paediatric < 18 years**	Seizure, generalized onset with or without secondary generalization, lasting < 5 minutes with < 24 hours post ictal state	Seizure, generalized onset with or without secondary generalization, lasting 5 – 20 minutes with < 24 hours post ictal state	Seizure, generalized onset with or without secondary generalization, lasting > 20 minutes	Seizure, generalized onset with or without secondary generalization, requiring intubation and sedation
Syncope (not associated with a procedure)	NA	Present	NA	NA
Vertigo	Vertigo causing no or minimal interference with usual social & functional activities	Vertigo causing greater than minimal interference with usual social & functional activities	Vertigo causing inability to perform usual social & functional activities	Disabling vertigo causing inability to perform basic self-care functions
**RESPIRATORY**				
Bronchospasm (acute)	FEV1 or peak flow reduced to 70 – 80%	FEV1 or peak flow 50 – 69%	FEV1 or peak flow 25 – 49%	Cyanosis OR FEV1 or peak flow < 25% OR Intubation
Dyspnea or respiratory distress				
**Adult ≥ 14 years**	Dyspnea on exertion with no or minimal interference with usual social & functional activities	Dyspnea on exertion causing greater than minimal interference with usual social & functional activities	Dyspnea at rest causing inability to perform usual social & functional activities	Respiratory failure with ventilatory support indicated
**Paediatric < 14 years**	Wheezing OR minimal increase in respiratory rate for age	Nasal flaring OR Intercostal retractions OR Pulse oximetry 90 – 95%	Dyspnea at rest causing inability to perform usual social & functional activities OR Pulse oximetry < 90%	Respiratory failure with ventilatory support indicated

CLINICAL				
**PARAMETER**	**GRADE 1 MILD**	**GRADE 2 MODERATE**	**GRADE 3 SEVERE**	**GRADE 4 POTENTIALLY LIFE-THREATENING**
**MUSCULOSKELETAL**				
Arthralgia<br>See also Arthritis	Joint pain causing no or minimal interference with usual social & functional activities	Joint pain causing greater than minimal interference with usual social & functional activities	Joint pain causing inability to perform usual social & functional activities	Disabling joint pain causing inability to perform basic self-care functions
Arthritis<br>See also Arthralgia	Stiffness or joint swelling causing no or minimal interference with usual social & functional activities	Stiffness or joint swelling causing greater than minimal interference with usual social & functional activities	Stiffness or joint swelling causing inability to perform usual social & functional activities	Disabling joint stiffness or swelling causing inability to perform basic self-care functions
Bone Mineral Loss				
**Adult ≥ 21 years**	BMD t-score -2.5 to -1.0	BMD t-score < -2.5	Pathological fracture (including loss of vertebral height)	Pathologic fracture causing life-threatening consequences
**Paediatric < 21 years**	BMD z-score -2.5 to -1.0	BMD z-score < -2.5	Pathological fracture (including loss of vertebral height)	Pathologic fracture causing life-threatening consequences
Myalgia (<u>non-injection site</u>)	Muscle pain causing no or minimal interference with usual social & functional activities	Muscle pain causing greater than minimal interference with usual social & functional activities	Muscle pain causing inability to perform usual social & functional activities	Disabling muscle pain causing inability to perform basic self-care functions
Osteonecrosis	NA	Asymptomatic with radiographic findings AND No operative intervention indicated	Symptomatic bone pain with radiographic findings OR Operative intervention indicated	Disabling bone pain with radiographic findings causing inability to perform basic self-care functions
**GENITOURINARY**				
Cervicitis (<u>symptoms</u>)<br>(For use in studies evaluating topical study agents)<br>For other cervicitis see Infection: Infection (any other than HIV infection)	Symptoms causing no or minimal interference with usual social & functional activities	Symptoms causing greater than minimal interference with usual social & functional activities	Symptoms causing inability to perform usual social & functional activities	Symptoms causing inability to perform basic self-care functions
Cervicitis (<u>clinical exam</u>)<br>(For use in studies evaluating topical study agents)<br>For other cervicitis see Infection: Infection (any other than HIV infection)	Minimal cervical abnormalities on examination (erythema, mucopurulent discharge, or friability) OR Epithelial disruption < 25% of total surface	Moderate cervical abnormalities on examination (erythema, mucopurulent discharge, or friability) OR Epithelial disruption of 25 – 49% total surface	Severe cervical abnormalities on examination (erythema, mucopurulent discharge, or friability) OR Epithelial disruption 50 – 75% total surface	Epithelial disruption > 75% total surface

CLINICAL				
**PARAMETER**	**GRADE 1 MILD**	**GRADE 2 MODERATE**	**GRADE 3 SEVERE**	**GRADE 4 POTENTIALLY LIFE-THREATENING**
Inter-menstrual bleeding (IMB)	Spotting observed by participant OR Minimal blood observed during clinical or colposcopic examination	Inter-menstrual bleeding not greater in duration or amount than usual menstrual cycle	Inter-menstrual bleeding greater in duration or amount than usual menstrual cycle	Hemorrhage with life-threatening hypotension OR Operative intervention indicated
Urinary tract obstruction (e.g., stone)	NA	Signs or symptoms of urinary tract obstruction without hydronephrosis or renal dysfunction	Signs or symptoms of urinary tract obstruction with hydronephrosis or renal dysfunction	Obstruction causing life-threatening consequences
Vulvovaginitis (symptoms)<br>(Use in studies evaluating topical study agents)<br>For other vulvovaginitis see Infection: Infection (any other than HIV infection)	Symptoms causing no or minimal interference with usual social & functional activities	Symptoms causing greater than minimal interference with usual social & functional activities	Symptoms causing inability to perform usual social & functional activities	Symptoms causing inability to perform basic self-care functions
Vulvovaginitis (clinical exam)<br>(Use in studies evaluating topical study agents)<br>For other vulvovaginitis see Infection: Infection (any other than HIV infection)	Minimal vaginal abnormalities on examination OR Epithelial disruption < 25% of total surface	Moderate vaginal abnormalities on examination OR Epithelial disruption of 25 - 49% total surface	Severe vaginal abnormalities on examination OR Epithelial disruption 50 - 75% total surface	Vaginal perforation OR Epithelial disruption > 75% total surface
**OCULAR/VISUAL**				
Uveitis	Asymptomatic but detectable on exam	Symptomatic anterior uveitis OR Medical intervention indicated	Posterior or pan-uveitis OR Operative intervention indicated	Disabling visual loss in affected eye(s)
Visual changes (from baseline)	Visual changes causing no or minimal interference with usual social & functional activities	Visual changes causing greater than minimal interference with usual social & functional activities	Visual changes causing inability to perform usual social & functional activities	Disabling visual loss in affected eye(s)
**ENDOCRINE/METABOLIC**				
Abnormal fat accumulation (e.g., back of neck, breasts, abdomen)	Detectable by study participant (or by caregiver for young children and disabled adults)	Detectable on physical exam by health care provider	Disfiguring OR Obvious changes on casual visual inspection	NA
Diabetes mellitus	NA	New onset without need to initiate medication OR Modification of current medications to regain glucose control	New onset with initiation of medication indicated OR Diabetes uncontrolled despite treatment modification	Life-threatening consequences (e.g., ketoacidosis, hyperosmolar non-ketotic coma)

CLINICAL

PARAMETER	GRADE 1<br>MILD	GRADE 2<br>MODERATE	GRADE 3<br>SEVERE	GRADE 4<br>POTENTIALLY LIFE-THREATENING
Gynecomastia	Detectable by study participant or caregiver (for young children and disabled adults)	Detectable on physical exam by health care provider	Disfiguring OR Obvious on casual visual inspection	NA
Hyperthyroidism	Asymptomatic	Symptomatic causing greater than minimal interference with usual social & functional activities OR Thyroid suppression therapy indicated	Symptoms causing inability to perform usual social & functional activities OR Uncontrolled despite treatment modification	Life-threatening consequences (e.g., thyroid storm)
Hypothyroidism	Asymptomatic	Symptomatic causing greater than minimal interference with usual social & functional activities OR Thyroid replacement therapy indicated	Symptoms causing inability to perform usual social & functional activities OR Uncontrolled despite treatment modification	Life-threatening consequences (e.g., myxedema coma)
Lipoatrophy<br>(e.g., fat loss from the face, extremities, buttocks)	Detectable by study participant (or by caregiver for young children and disabled adults)	Detectable on physical exam by health care provider	Disfiguring OR Obvious on casual visual inspection	NA

LABORATORY

PARAMETER	GRADE 1<br>MILD	GRADE 2<br>MODERATE	GRADE 3<br>SEVERE	GRADE 4<br>POTENTIALLY LIFE-THREATENING
**HAEMATOLOGY**	***Standard International Units are listed in italics***			
Absolute CD4+ count **– Adult and Paediatric > 13 years** (HIV <u>NEGATIVE</u> ONLY)	300 – 400/mm^3<br>*300 – 400/µL*	200 – 299/mm^3<br>*200 – 299/µL*	100 – 199/mm^3<br>*100 – 199/µL*	< 100/mm^3<br>*< 100/µL*
Absolute lymphocyte count **– Adult and Paediatric > 13 years** (HIV <u>NEGATIVE</u> ONLY)	600 – 650/mm^3<br>*0.600 x 10^9 – 0.650 x 10^9/L*	500 – 599/mm^3<br>*0.500 x 10^9 – 0.599 x 10^9/L*	350 – 499/mm^3<br>*0.350 x 10^9 – 0.499 x 10^9/L*	< 350/mm^3<br>*< 0.350 x 10^9/L*
Absolute neutrophil count (ANC) [9, 44]				
**Adult and Paediatric, > 7 days**	750 –< 1,000/mm^3<br>*0.75 x 10^9 – <1.0. x 10^9/L*	500 – 749/mm^3<br>*0.5 x 10^9 – 0.749 x 10^9/L*	250 – 499/mm^3<br>*0.25 x 10^9 – 0.499 x 10^9/L*	< 250/mm^3<br>*< 0.250 x 10^9/L*
**Infant*†, 2 – ≤ 7 days**	1,250 – 1,500/mm^3<br>*1.250 x 10^9 – 1.500 x 10^9/L*	1,000 – 1,249/mm^3<br>*1.000 x 10^9 – 1.249 x 10^9/L*	750 – 999/mm^3<br>*0.750 x 10^9 – 0.999 x 10^9/L*	< 750/mm^3<br>*< 0.750 x 10^9/L*
**Infant*†, 1 day**	4,000 – 5,000/mm^3<br>*4.000 x 10^9 – 5.000 x 10^9/L*	3,000 – 3,999/mm^3<br>*3.000 x 10^9 – 3.999 x10^9/L*	1,500 – 2,999/mm^3<br>*1.500 x 10^9 – 2.999 x 10^9/L*	< 1,500/mm^3<br>*< 1.500 x 10^9/L*

CLINICAL					
**PARAMETER**		**GRADE 1 MILD**	**GRADE 2 MODERATE**	**GRADE 3 SEVERE**	**GRADE 4 POTENTIALLY LIFE-THREATENING**
Fibrinogen, decreased		100 – 200 mg/dL<br>*1.00 – 2.00 g/L*<br>OR<br>0.75 – 0.99 x LLN	75 – 99 mg/dL<br>*0.75 – 0.99 g/L*<br>OR<br>0.50 – 0.74 x LLN	50 – 74 mg/dL<br>*0.50 – 0.74 g/L*<br>OR<br>0.25 – 0.49 x LLN	< 50 mg/dL<br>*< 0.50 g/L*<br>OR<br>< 0.25 x LLN<br>OR<br>Associated with gross bleeding
Haemoglobin (Hgb)					
	**Adult and Paediatric ≥ 57 days** (HIV POSITIVE ONLY)	8.5 – 10.0 g/dL<br>*1.32 – 1.55 mmol/L*	7.5 – 8.4 g/dL<br>*1.16 – 1.31 mmol/L*	6.50 – 7.4 g/dL<br>*1.01 – 1.15 mmol/L*	< 6.5 g/dL<br>*< 1.01 mmol/L*
	**Adult and Paediatric ≥ 57 days** (HIV NEGATIVE ONLY)	10.0 – 10.9 g/dL<br>*1.55 – 1.69 mmol/L*<br>OR<br>Any decrease<br>2.5 – 3.4 g/dL<br>*0.39 – 0.53 mmol/L*	9.0 – 9.9 g/dL<br>*1.40 – 1.54 mmol/L*<br>OR<br>Any decrease<br>3.5 – 4.4 g/dL<br>*0.54 – 0.68 mmol/L*	7.0 – 8.9 g/dL<br>*1.09 – 1.39 mmol/L*<br>OR<br>Any decrease<br>≥ 4.5 g/dL<br>*≥ 0.69 mmol/L*	< 7.0 g/dL<br>*< 1.09 mmol/L*
	**Infant*†, 36 – 56 days** (HIV POSITIVE OR NEGATIVE)	8.5 – 9.4 g/dL<br>*1.32 – 1.46 mmol/L*	7.0 – 8.4 g/dL<br>*1.09 – 1.31 mmol/L*	6.0 – 6.9 g/dL<br>*0.93 – 1.08 mmol/L*	< 6.00 g/dL<br>*< 0.93 mmol/L*
	**Infant*†, 22 – 35 days** (HIV POSITIVE OR NEGATIVE)	9.5 – 10.5 g/dL<br>*1.47 – 1.63 mmol/L*	8.0 – 9.4 g/dL<br>*1.24 – 1.46 mmol/L*	7.0 – 7.9 g/dL<br>*1.09 – 1.23 mmol/L*	< 7.00 g/dL<br>*< 1.09 mmol/L*
	**Infant*†, 1 – 21 days** (HIV POSITIVE OR NEGATIVE)	12.0 – 13.0 g/dL<br>*1.86 – 2.02 mmol/L*	10.0 – 11.9 g/dL<br>*1.55 – 1.85 mmol/L*	9.0 – 9.9 g/dL<br>*1.40 – 1.54 mmol/L*	< 9.0 g/dL<br>*< 1.40 mmol/L*
International Normalized Ratio of prothrombin time (INR)		1.1 – 1.5 x ULN	1.6 – 2.0 x ULN	2.1 – 3.0 x ULN	> 3.0 x ULN
Methemoglobin		5.0 – 10.0%	10.1 – 15.0%	15.1 – 20.0%	> 20.0%
Prothrombin Time (PT)		1.1 – 1.25 x ULN	1.26 – 1.50 x ULN	1.51 – 3.00 x ULN	> 3.00 x ULN
Partial Thromboplastin Time (PTT)		1.1 – 1.66 x ULN	1.67 – 2.33 x ULN	2.34 – 3.00 x ULN	> 3.00 x ULN
Platelets, decreased		100,000 – 124,999/mm^3<br>*100.000 x 10^9 – 124.999 x 10^9/L*	50,000 – 99,999/mm^3<br>*50.000 x 10^9 – 99.999 x 10^9/L*	25,000 – 49,999/mm^3<br>*25.000 x 10^9 – 49.999 x 10^9/L*	< 25,000/mm^3<br>*< 25.000 x 10^9/L*
WBC, decreased		2,000 – 2,500/mm^3<br>*2.000 x 10^9 – 2.500 x 10^9/L*	1,500 – 1,999/mm^3<br>*1.500 x 10^9 – 1.999 x 10^9/L*	1,000 – 1,499/mm^3<br>*1.000 x 10^9 – 1.499 x 10^9/L*	< 1,000/mm^3<br>*< 1.000 x 10^9/L*
**CHEMISTRIES**		***Standard International Units are listed in italics***			
Acidosis		NA	pH < normal, but ≥ 7.3	pH < 7.3 without life-threatening consequences	pH < 7.3 with life-threatening consequences
Albumin, serum, low		3.0 g/dL – < LLN<br>*30 g/L – < LLN*	2.0 – 2.9 g/dL<br>*20 – 29 g/L*	< 2.0 g/dL<br>*< 20 g/L*	NA
Alkaline Phosphatase		1.25 – 2.5 x ULN†	2.6 – 5.0 x ULN†	5.1 – 10.0 x ULN†	> 10.0 x ULN†

CLINICAL				
**PARAMETER**	**GRADE 1<br>MILD**	**GRADE 2<br>MODERATE**	**GRADE 3<br>SEVERE**	**GRADE 4<br>POTENTIALLY<br>LIFE-THREATENING**
Alkalosis	NA	pH > normal, but ≤ 7.5	pH > 7.5 without life-threatening consequences	pH > 7.5 with life-threatening consequences
ALT (SGPT)	1.25 – 2.5 x ULN	2.6 – 5.0 x ULN	5.1 – 10.0 x ULN	> 10.0 x ULN
AST (SGOT)	1.25 – 2.5 x ULN	2.6 – 5.0 x ULN	5.1 – 10.0 x ULN	> 10.0 x ULN
Bicarbonate, serum, low	16.0 mEq/L – < LLN<br>*16.0 mmol/L – < LLN*	11.0 – 15.9 mEq/L<br>*11.0 – 15.9 mmol/L*	8.0 – 10.9 mEq/L<br>*8.0 – 10.9 mmol/L*	< 8.0 mEq/L<br>*< 8.0 mmol/L*
Bilirubin (Total)				
**Adult and Paediatric > 14 days**	1.1 – 1.5 x ULN	1.6 – 2.5 x ULN	2.6 – 5.0 x ULN	> 5.0 x ULN
**Infant[*,†], ≤ 14 days** (non-hemolytic)	NA	20.0 – 25.0 mg/dL<br>*342 – 428 µmol/L*	25.1 – 30.0 mg/dL<br>*429 – 513 µmol/L*	> 30.0 mg/dL<br>*> 513.0 µmol/L*
**Infant[*,†], ≤ 14 days** (hemolytic)	NA	NA	20.0 – 25.0 mg/dL<br>*342 – 428 µmol/L*	> 25.0 mg/dL<br>*> 428 µmol/L*
Calcium, serum, high (corrected for albumin)				
**Adult and Paediatric ≥ 7 days**	10.6 – 11.5 mg/dL<br>*2.65 – 2.88 mmol/L*	11.6 – 12.5 mg/dL<br>*2.89 – 3.13 mmol/L*	12.6 – 13.5 mg/dL<br>*3.14 – 3.38 mmol/L*	> 13.5 mg/dL<br>*> 3.38 mmol/L*
**Infant[*,†], < 7 days**	11.5 – 12.4 mg/dL<br>*2.88 – 3.10 mmol/L*	12.5 – 12.9 mg/dL<br>*3.11 – 3.23 mmol/L*	13.0 – 13.5 mg/dL<br>*3.245 – 3.38 mmol/L*	> 13.5 mg/dL<br>*> 3.38 mmol/L*
Calcium, serum, low (corrected for albumin)				
**Adult and Paediatric ≥ 7 days**	7.8 – 8.4 mg/dL<br>*1.95 – 2.10 mmol/L*	7.0 – 7.7 mg/dL<br>*1.75 – 1.94 mmol/L*	6.1 – 6.9 mg/dL<br>*1.53 – 1.74 mmol/L*	< 6.1 mg/dL<br>*< 1.53 mmol/L*
**Infant[*,†], < 7 days**	6.5 – 7.5 mg/dL<br>*1.63 – 1.88 mmol/L*	6.0 – 6.4 mg/dL<br>*1.50 – 1.62 mmol/L*	5.50 – 5.90 mg/dL<br>*1.38 – 1.51 mmol/L*	< 5.50 mg/dL<br>*< 1.38 mmol/L*
Cardiac troponin I (cTnI)	NA	NA	NA	Levels consistent with myocardial infarction or unstable angina as defined by the manufacturer
Cardiac troponin T (cTnT)	NA	NA	NA	≥ 0.20 ng/mL<br>OR<br>Levels consistent with myocardial infarction or unstable angina as defined by the manufacturer
Cholesterol (fasting)				
**Adult ≥ 18 years**	200 – 239 mg/dL<br>*5.18 – 6.19 mmol/L*	240 – 300 mg/dL<br>*6.20 – 7.77 mmol/L*	> 300 mg/dL<br>*> 7.77 mmol/L*	NA
**Paediatric < 18 years**	170 – 199 mg/dL<br>*4.40 – 5.15 mmol/L*	200 – 300 mg/dL<br>*5.16 – 7.77 mmol/L*	> 300 mg/dL<br>*> 7.77 mmol/L*	NA
Creatine Kinase	3.0 – 5.9 x ULN[†]	6.0 – 9.9 x ULN[†]	10.0 – 19.9 x ULN[†]	≥ 20.0 x ULN[†]
Creatinine	1.1 – 1.3 x ULN[†]	1.4 – 1.8 x ULN[†]	1.9 – 3.4 x ULN[†]	≥ 3.5 x ULN[†]
Glucose, serum, high				
Nonfasting	116 – 160 mg/dL<br>*6.44 – 8.88 mmol/L*	161 – 250 mg/dL<br>*8.89 – 13.88 mmol/L*	251 – 500 mg/dL<br>*13.89 – 27.75 mmol/L*	> 500 mg/dL<br>*> 27.75 mmol/L*

CLINICAL				
**PARAMETER**	**GRADE 1 MILD**	**GRADE 2 MODERATE**	**GRADE 3 SEVERE**	**GRADE 4 POTENTIALLY LIFE-THREATENING**
Fasting	110 – 125 mg/dL<br>*6.11 – 6.94 mmol/L*	126 – 250 mg/dL<br>*6.95 – 13.88 mmol/L*	251 – 500 mg/dL<br>*13.89 – 27.75 mmol/L*	> 500 mg/dL<br>*> 27.75 mmol/L*
Glucose, serum, low				
**Adult and Paediatric ≥ 1 month**	55 – 64 mg/dL<br>*3.05 – 3.55 mmol/L*	40 – 54 mg/dL<br>*2.22 – 3.06 mmol/L*	30 – 39 mg/dL<br>*1.67 – 2.23 mmol/L*	< 30 mg/dL<br>*< 1.67 mmol/L*
**Infant*†, < 1 month**	50 – 54 mg/dL<br>*2.78 – 3.00 mmol/L*	40 – 49 mg/dL<br>*2.22 – 2.77 mmol/L*	30 – 39 mg/dL<br>*1.67 – 2.21 mmol/L*	< 30 mg/dL<br>*< 1.67 mmol/L*
Lactate	< 2.0 x ULN without acidosis	≥ 2.0 x ULN without acidosis	Increased lactate with pH < 7.3 without life-threatening consequences	Increased lactate with pH < 7.3 with life-threatening consequences
LDL cholesterol (fasting)				
**Adult ≥ 18 years**	130 – 159 mg/dL<br>*3.37 – 4.12 mmol/L*	160 – 190 mg/dL<br>*4.13 – 4.90 mmol/L*	≥ 190 mg/dL<br>*≥ 4.91 mmol/L*	NA
**Paediatric > 2 - < 18 years**	110 – 129 mg/dL<br>*2.85 – 3.34 mmol/L*	130 – 189 mg/dL<br>*3.35 – 4.90 mmol/L*	≥ 190 mg/dL<br>*≥ 4.91 mmol/L*	NA
Lipase	1.1 – 1.5 x ULN	1.6 – 3.0 x ULN	3.1 – 5.0 x ULN	> 5.0 x ULN
Magnesium, serum, low	1.2 – 1.4 mEq/L<br>*0.60 – 0.70 mmol/L*	0.9 – 1.1 mEq/L<br>*0.45 – 0.59 mmol/L*	0.6 – 0.8 mEq/L<br>*0.30 – 0.44 mmol/L*	< 0.60 mEq/L<br>*< 0.30 mmol/L*
Pancreatic amylase	1.1 – 1.5 x ULN	1.6 – 2.0 x ULN	2.1 – 5.0 x ULN	> 5.0 x ULN
Phosphate, serum, low				
**Adult and Paediatric > 14 years**	2.5 mg/dL – < LLN<br>*0.81 mmol/L – < LLN*	2.0 – 2.4 mg/dL<br>*0.65 – 0.80 mmol/L*	1.0 – 1.9 mg/dL<br>*0.32 – 0.64 mmol/L*	< 1.00 mg/dL<br>*< 0.32 mmol/L*
**Paediatric 1 year – 14 years**	3.0 – 3.5 mg/dL<br>*0.97 – 1.13 mmol/L*	2.5 – 2.9 mg/dL<br>*0.81 – 0.96 mmol/L*	1.5 – 2.4 mg/dL<br>*0.48 – 0.80 mmol/L*	< 1.50 mg/dL<br>*< 0.48 mmol/L*
**Paediatric < 1 year**	3.5 – 4.5 mg/dL<br>*1.13 – 1.45 mmol/L*	2.5 – 3.4 mg/dL<br>*0.81 – 1.12 mmol/L*	1.5 – 2.4 mg/dL<br>*0.48 – 0.80 mmol/L*	< 1.50 mg/dL<br>*< 0.48 mmol/L*
Potassium, serum, high	5.6 – 6.0 mEq/L<br>*5.6 – 6.0 mmol/L*	6.1 – 6.5 mEq/L<br>*6.1 – 6.5 mmol/L*	6.6 – 7.0 mEq/L<br>*6.6 – 7.0 mmol/L*	> 7.0 mEq/L<br>*> 7.0 mmol/L*
Potassium, serum, low	3.0 – 3.4 mEq/L<br>*3.0 – 3.4 mmol/L*	2.5 – 2.9 mEq/L<br>*2.5 – 2.9 mmol/L*	2.0 – 2.4 mEq/L<br>*2.0 – 2.4 mmol/L*	< 2.0 mEq/L<br>*< 2.0 mmol/L*
Sodium, serum, high	146 – 150 mEq/L<br>*146 – 150 mmol/L*	151 – 154 mEq/L<br>*151 – 154 mmol/L*	155 – 159 mEq/L<br>*155 – 159 mmol/L*	≥ 160 mEq/L<br>*≥ 160 mmol/L*
Sodium, serum, low	130 – 135 mEq/L<br>*130 – 135 mmol/L*	125 – 129 mEq/L<br>*125 – 129 mmol/L*	121 – 124 mEq/L<br>*121 – 124 mmol/L*	≤ 120 mEq/L<br>*≤ 120 mmol/L*
Triglycerides (fasting)	NA	500 – 750 mg/dL<br>*5.65 – 8.48 mmol/L*	751 – 1,200 mg/dL<br>*8.49 – 13.56 mmol/L*	> 1,200 mg/dL<br>*> 13.56 mmol/L*
Uric acid	7.5 – 10.0 mg/dL<br>*0.45 – 0.59 mmol/L*	10.1 – 12.0 mg/dL<br>*0.60 – 0.71 mmol/L*	12.1 – 15.0 mg/dL<br>*0.72 – 0.89 mmol/L*	> 15.0 mg/dL<br>*> 0.89 mmol/L*
**URINALYSIS**	***Standard International Units are listed in italics***			
Hematuria (microscopic)	6 – 10 RBC/HPF	> 10 RBC/HPF	Gross, with or without clots OR with RBC casts	Transfusion indicated
Proteinuria, random collection	1 +	2 – 3 +	4 +	NA
Proteinuria, 24 hour collection				

CLINICAL				
**PARAMETER**	**GRADE 1 MILD**	**GRADE 2 MODERATE**	**GRADE 3 SEVERE**	**GRADE 4 POTENTIALLY LIFE-THREATENING**
**Adult and Paediatric ≥ 10 years**	200 – 999 mg/24 h<br>*0.200 – 0.999 g/d*	1,000 – 1,999 mg/24 h<br>*1.000 – 1.999 g/d*	2,000 – 3,500 mg/24 h<br>*2.000 – 3.500 g/d*	> 3,500 mg/24 h<br>*> 3.500 g/d*
**Paediatric > 3 mo - < 10 years**	201 – 499 mg/m^2/24 h<br>*0.201 – 0.499 g/d*	500 – 799 mg/m^2/24 h<br>*0.500 – 0.799 g/d*	800 – 1,000 mg/m^2/24 h<br>*0.800 – 1.000 g/d*	> 1,000 mg/ m^2/24 h<br>*> 1.000 g/d*

*Values are for term infants. †Use age and sex appropriate values (e.g., bilirubin), including preterm infants.

Appendix 4: WHO Staging

Paediatric WHO clinical staging for HIV/AIDS for infants and children with established HIV infection[1]

Clinical Stage 1

Asymptomatic
Persistent generalized lymphadenopathy

Clinical Stage 2

Unexplained persistent hepatosplenomegaly
Papular pruritic eruptions
Extensive wart virus infection
Extensive molluscum contagiosum
Recurrent oral ulcerations
Unexplained persistent parotid enlargement
Lineal gingival erythema
Herpes zoster
Recurrent or chronic respiratory tract infections (otitis media, otorrhoea, sinusitis or tonsillitis)
Fungal nail infections

Clinical Stage 3

Unexplained moderate malnutrition not adequately responding to standard therapy
Unexplained persistent diarrhoea (14 days or more)
Unexplained persistent fever (above 37.5 ^{o}C intermittent or constant, for longer than one month)
Persistent oral candidiasis (after first 6- 8 weeks of life)
Oral hairy leukoplakia
Acute necrotizing ulcerative gingivitis or periodontitis
Lymph node tuberculosis
Pulmonary tuberculosis
Severe recurrent bacterial pneumonia
Symptomatic lymphoid interstitial pneumonitis
Chronic HIV-associated lung disease including brochiectasis
Unexplained anaemia (<8g/dl), neutropenia (<500/mm^3) and/or chronic thrombocytopenia (<50 000/ mm^3)

Clinical Stage 4

Unexplained severe wasting, stunting or severe malnutrition not responding to standard therapy
Pneumocystis pneumonia
Recurrent severe presumed bacterial infections (e.g. empyema, pyomyositis, bone or joint infection, meningitis, but excluding pneumonia)
Chronic herpes simplex infection (orolabial or cutaneous of more than one month's duration or visceral at any site)
Extrapulmonary tuberculosis
Kaposi sarcoma
Oesophageal candidiasis (or candidiasis of trachea, bronchi or lungs)
Central nervous system toxoplasmosis (after one month of life)
HIV encephalopathy
Cytomegalovirus (CMV) infection; retinitis or CMV infection affecting another organ, with onset at age over one month
Extrapulmonary cryptococcosis (including meningitis)
Disseminated endemic mycosis (extrapulmonary histoplasmosis, coccidiomycosis)
Chronic cryptosporidiosis
Chronic isosporiasis
Disseminated non-tuberculous mycobacterial infection
Cerebral or B-cell non-Hodgkin lymphoma
Progressive multifocal leukoencephalopathy
Symptomatic HIV-associated nephropathy or HIV-associated cardiomyopathy

[1] All clinical events or conditions referred to are described in section below

Presumptive and definitive criteria for recognizing HIV/AIDS-related clinical events in infants and children with established HIV infection

Clinical event	Clinical diagnosis	Definitive diagnosis
**Clinical Stage 1**		
Asymptomatic	No HIV related symptoms reported and no clinical signs on examination.	Not applicable.
Persistent generalized lymphadenopathy	Persistent swollen or enlarged lymph nodes >1 cm at two or more non-contiguous sites (excluding inguinal) without known cause.	Clinical diagnosis
**Clinical Stage 2**		
Unexplained persistent hepatosplenomegaly	Enlarged liver and spleen without obvious cause.	Clinical diagnosis
Papular pruritic eruptions	Papular pruritic vesicular lesions. Also common in uninfected children: scabies and insect bites should be excluded.	Clinical diagnosis
Extensive wart virus infection	Characteristic warty skin lesions; small fleshy grainy bumps, often rough, flat on sole of feet (plantar warts); facial, more than 5% of body area or disfiguring.	Clinical diagnosis
Extensive molluscum contagiosum infection	Characteristic skin lesions: small flesh-coloured, pearly or pink, dome-shaped or umbilicated growths, may be inflamed or red; facial, more than 5% of body area or disfiguring. Giant molloscum may indicate more advanced immunodeficiency	Clinical diagnosis
Fungal nail infections	Fungal paronychia (painful, red and swollen nail bed) or onycholysis (painless separation of the nail from the nail bed.) Proximal white subungual onchomycosis is uncommon without immunodeficiency	Clinical diagnosis
Recurrent oral ulcerations	Current event plus at least one previous episode in past six months. Aphthous ulceration, typically with a halo of inflammation & yellow-grey pseudomembrane.	Clinical diagnosis
Unexplained persistent parotid enlargement	Asymptomatic bilateral swelling that may spontaneously resolve and recur, in absence of other known cause, usually painless	Clinical diagnosis
Lineal gingival erythema	Erythematous band that follows the contour of the free gingival line; may be associated with spontaneous bleeding	Clinical diagnosis
Herpes zoster	Painful rash with fluid-filled blisters, dermatomal distribution, can be haemorrhagic on erythematous background, and can become large and confluent. Does not cross the midline	Clinical diagnosis
Recurrent upper respiratory tract infection	Current event with at least one episode in past 6 months. Symptom complex; fever with unilateral face pain and nasal discharge (sinusitis) or painful swollen eardrum (otitis media), sore throat with productive cough (bronchitis), sore throat (pharyngitis) and barking croup-like cough (LTB). Persistent or recurrent ear discharge.	Clinical diagnosis
**Clinical Stage 3**		
Unexplained moderate malnutrition	Weight loss: low weight-for-age, up to −2 standard deviations from the mean, not explained by poor or inadequate feeding and or other infections, and not	Confirmed by documented loss of body weight of −2 standard deviations from the mean, failure to gain weight on standard

Clinical event	Clinical diagnosis	Definitive diagnosis
	adequately responding to standard management.	management and no other cause identified during investigation.
Unexplained persistent diarrhoea	Unexplained persistent (14 days or more) diarrhoea (loose or watery stool, three or more times daily), not responding to standard treatment.	Confirmed by stools observed and documented as unformed. Culture and microscopy reveal no pathogens.
Unexplained persistent fever (>37.5°C intermittent or constant for longer than one month)	Reports of fever or night sweats for longer than one month, either intermittent or constant, with reported lack of response to antibiotics or antimalarial agents. No other obvious foci of disease reported or found on examination. Malaria must be excluded in malarious areas.	Confirmed by documented fever of >37.5°C with negative blood culture, negative malaria slide and normal or unchanged chest X-ray, and no other obvious foci of disease.
Oral candidiasis<br>(after first 6-8 weeks of life)	Persistent or recurring creamy white to yellow soft small plaques which can be scraped off (pseudomembranous), or red patches on tongue, palate or lining of mouth, usually painful or tender (erythematous form).	Confirmed by microscopy or culture.
Oral hairy leukoplakia	Fine small linear patches on lateral borders of tongue, generally bilaterally, which do not scrape off.	Clinical diagnosis
Lymph node tuberculosis	Non acute, painless "cold" enlargement of peripheral lymph nodes, localized to one region. May have draining sinuses. Response to standard anti-tuberculosis treatment in one month.	Confirmed by histology or fine needle aspirate for Ziehl-Nielsen stain or culture.
Pulmonary tuberculosis	Nonspecific symptoms, e.g. chronic cough, fever, night sweats, anorexia and weight loss. In the older child also productive cough and haemoptysis. History of contact with adults with smear-positive pulmonary tuberculosis. No response to standard broad-spectrum antibiotic treatment.	Confirmed by one or more sputum positive smear for acid-fast bacilli and/or radiographic abnormalities consistent with active tuberculosis and/or culture-positive for *Mycobacterium*.
Severe recurrent bacterial pneumonia	Cough with fast breathing, chest indrawing, nasal flaring, wheezing, and grunting. Crackles or consolidation on auscultation. Responds to course of antibiotics. Current episode plus one or more in previous 6 months.	Confirmed by isolation of bacteria from appropriate clinical specimens (induced sputum, BAL, lung aspirate).
Acute necrotizing ulcerative gingivitis or stomatitis, or acute necrotizing ulcerative periodontitis	Severe pain, ulcerated gingival papillae, loosening of teeth, spontaneous bleeding, bad odour, and rapid loss of bone and/or soft tissue.	Clinical diagnosis
Symptomatic lymphocytic interstitial pneumonitis	No presumptive clinical diagnosis.	Diagnosed by chest X-ray: bilateral reticulonodular interstitial pulmonary infiltrates present for more than two months with no response to antibiotic treatment and no other pathogen found. Oxygen saturation persistently <90%. May present with cor pulmonale and may increased exercise-induced fatigue. Characteristic histology.
Chronic HIV-associated lung disease (including bronchiectasis)	History of cough productive of copious amounts of purulent sputum (bronchiectasis only), with or without clubbing, halitosis, and crepitations and/or wheezes on auscultation	Confirmed by chest X-ray: may show honeycomb appearance (small cysts) and/or persistent areas of opacification and/or widespread lung destruction, with fibrosis and loss of volume.
Unexplained anaemia (<8g/dl), or neutropaenia (<0.5 x 10^9 per litre) and/or chronic	No presumptive clinical diagnosis.	Diagnosed on laboratory testing, not explained by other non-HIV conditions, not responding to standard therapy with haematinics, antimalarial agents or

Clinical event	Clinical diagnosis	Definitive diagnosis
thrombocytopaenia (<50 x 10^9 per litre)		anthelmintic agents as outlined in WHO Integrated Management of Childhood Illness (IMCI) guidelines.
**Clinical Stage 4**		
Unexplained severe wasting, stunting or severe malnutrition not adequately responding to standard therapy	Persistent weight loss not explained by poor or inadequate feeding, other infections and not adequately responding in two weeks to standard therapy. Characterized by:<br>visible severe wasting of muscles, with or without oedema of both feet, and/or weight-for-height of –3 standard deviations from the mean, as defined by WHO ICMI guidelines.	Confirmed by documented weight loss of moer than -3 standard deviations from the mean with or without oedema
Pneumocystis pneumonia	Dry cough, progressive difficulty in breathing, cyanosis, tachypnoea and fever; chest indrawing or stridor. (Severe or very severe pneumonia as in WHO IMCI guidelines). Usually rapid onset especially in infants under six months of age. Response to high-dose co-trimoxazole with or without prednisolone. Chest X-ray shows typical bilateral perihilar diffuse infiltrates	Confirmed by: cytology or immunofluorescent microscopy of induced sputum or BAL or histology of lung tissue.
Recurrent severe bacterial infection, e.g. empyema, pyomyositis, bone or joint infection or meningitis but excluding pneumonia	Fever accompanied by specific symptoms or signs that localize infection. Responds to antibiotics. Current episode plus one or more in previous 6 months	Confirmed by culture of appropriate clinical specimen.
Chronic herpes simplex infection; (orolabial or cutaneous of more than one month's duration or visceral at any site)	Severe and progressive painful orolabial, genital, or anorectal lesions caused by HSV infection present for more than one month.	Confirmed by culture and/or histology
Oesophageal candidiasis<br>(or candidiasis of trachea, bronchi or lungs).	Difficulty in swallowing or pain on swallowing (food and fluids). In young children, suspect particularly if oral *Candida* observed and food refusal occurs and/or difficulties or crying when feeding.	Confirmed by macroscopic appearance at endoscopy, microscopy of specimen from tissue or macroscopic appearance at bronchoscopy or histology.
Extrapulmonary or disseminated tuberculosis	Systemic illness usually with prolonged fever, night sweats, and weight loss. Clinical features of organs involved, e.g. sterile pyuria, pericarditis, ascites, pleural effusion, meningitis, arthritis or orchiti, pericardial or abdominal	Confirmed by positive microscopy showing acid-fast bacilli or culture of *Mycobacterium* TB from blood or other relevant specimen except sputum or BAL. Biopsy and histology.
Kaposi sarcoma	Typical appearance in skin or oropharynx of persistent, initially flat, patches with a pink or blood-bruise colour, skin lesions that usually develop into nodules.	Not required but may be confirmed by:<br>- typical red-purple lesions seen on bronchoscopy or endoscopy;<br>- dense masses in lymph nodes, viscera or lungs by palpation or radiology; and<br>- histology.
CMV retinitis or CMV infection affecting another organ, with onset at age over 1 month.	Retinitis only.<br>CMV retinitis may be diagnosed by experienced clinicians: typical eye lesions on serial fundoscopic examination; discrete patches of retinal whitening with distinct borders, spreading centrifugally, often following blood vessels, associated with retinal vasculitis, haemorrhage and necrosis.	Definitive diagnosis required for other sites. Histology. Cerebrospinal fluid polymerase chain reaction

Clinical event	Clinical diagnosis	Definitive diagnosis
CNS toxoplasmosis with onset at age over 1 month.	Fever, headache, focal neurological signs, convulsions. Usually responds within 10 days to specific therapy.	Not required but confirmed by computed tomography (CT) scan (or other neuroimaging) showing single or multiple lesions with mass effect or enhancing with contrast.
Extrapulmonary cryptococcosis (including meningitis)	Meningitis: usually subacute, fever with increasing severe headache, meningism, confusion and behavioural changes that respond to cryptococcal therapy.	Confirmed by CSF microscopy (India ink or Gram stain), serum or CSF CRAG test or culture.
HIV encephalopathy	At least one of the following, progressing over at least two months in the absence of another illness:<br>- failure to attain, or loss of, developmental milestones, loss of intellectual ability;<br>OR<br>- progressive impaired brain growth demonstrated by stagnation of head circumference;<br>OR<br>- acquired symmetric motor deficit accompanied by two or more of the following: paresis, pathological reflexes, ataxia, gait disturbances.	Confirmed by neuroimaging (brain CT scan or MRI) demonstrating atrophy and basal ganglia calcification and excluding other causes.
Disseminated mycosis (coccidiomycosis, histoplasmosis, penicilliosis)	No presumptive clinical diagnosis.	Diagnosed by:<br>Histology: usually granuloma formation.<br>Isolation: antigen detection from affected tissue; culture or microscopy from clinical specimen or blood culture.
Disseminated mycobacteriosis,<br>other than TB	No presumptive clinical diagnosis.	Nonspecific clinical symptoms including progressive weight loss, fever, anaemia, night sweats, fatigue or diarrhoea; plus culture of atypical mycobacterial species from stool, blood, body fluid or other body tissue, excluding lung.
Chronic cryptosporidiosis	No presumptive clinical diagnosis.	Confirmed by cysts identified on modified Ziehl-Nielsen microscopic examination of unformed stool
Chronic *Isospora*	No presumptive clinical diagnosis.	Confirmed in children with chronic diarrhoea by microscopic examination.
Cerebral or B-cell non-Hodgkin lymphoma	No presumptive clinical diagnosis.	Diagnosed by CNS neuroimaging: at least one lesion with mass effect on brain scan; histology of relevant specimen
Progressive multi focal leukoencephalopathy	No presumptive clinical diagnosis.	Diagnosed by progressive nervous system disorder (cognitive dysfunction, gait or speech disorder, visual loss, limb weakness and cranial nerve palsies) together with hypodense white matter lesions on neuroimaging or positive polyomavirus Jacob Creutzfeldt PCR on cerebrospinal fluid
Symptomatic HIV-associated nephropathy	No presumptive clinical diagnosis.	Renal biopsy

Clinical event	Clinical diagnosis	Definitive diagnosis
Symptomatic HIV-associated cardiomyopathy	No presumptive clinical diagnosis.	Cardiomegaly and evidence of poor left ventricular function confirmed by echocardiography

PRESUMPTIVE DIAGNOSIS OF SEVERE HIV DISEASE IN INFANTS

Taken from: *WHO case definitions of HIV for surveillance and revised clinical staging and immunological classification of HIV-related disease in adults and children. 2007*

The presumptive diagnosis is designed for use where access to confirmatory diagnostic testing for HIV infection using virological or P24 Antigen for infants and children less than 18 months is not readily available. It is not recommended for use by clinical care providers who are not trained on ART, accredited or certified and experienced in HIV care, and must be accompanied by immediate efforts to confirm the HIV diagnosis with the best nationally or locally available test.

Clinical criteria for presumptive diagnosis of severe HIV disease among infants and children aged under 18 months in situations where virological testing is not available
A presumptive diagnosis of severe HIV disease should be made if:<br>• the infant is confirmed as HIV antibody-positive;<br>*and*<br>• diagnosis of any AIDS-indicator condition(s)[a] can be made;<br>*or*<br>• the infant is symptomatic with two or more of the following;<br>oral thrush[b];<br>severe pneumonia[b];<br>severe sepsis[b].<br>Other factors that support the diagnosis of severe HIV disease in an HIV-seropositive infant include:<br>• recent HIV-related maternal death or advanced HIV disease in the mother;<br>• CD4 <20%.c<br>Confirmation of the diagnosis of HIV infection should be sought as soon as possible.

[a] AIDS indicator conditions include some but not all HIV clinical stage 4 conditions seen in children such as Pneumocystis pneumonia,
oesophageal candidiasis, cryptococcal meningitis, cerebral toxoplasmosis, unexplained wasting or malnutrition.
[b] Defined in accordance with WHO Integrated Management of Childhood Illness guidelines:
• Oral thrush: Creamy white soft small plaques on red or normally coloured mucosa which can often be scraped off (pseudomembranous),
or red patches on tongue, palate or lining of mouth, usually painful or tender.
• Severe pneumonia: Cough or difficult breathing in a child with chest indrawing, stridor or any of the general danger signs
outlined in the WHO Integrated Management of Childhood Illness guidelines: that is lethargic or unconscious, not able to
drink or breastfeed, vomiting and presence or history of convulsions during current illness,.
• Severe sepsis: Fever or low body temperature in a young infant with any severe sign, such as rapid breathing, chest indrawing,
bulging fontanelle, lethargy, reduced movement, not feeding or sucking breast-milk, convulsions, stiff neck.
[c] It is unclear how often the CD4 count is lowered in these conditions in HIV-uninfected children.

Appendix 5: DART PREGNANCY MANUAL OF OPERATIONS

Pregnancy and birth

Logistics and linking with Obstetric Services

Women may come from different geographical locations and therefore referral to and liaison with a single maternity facility may not be possible. However, it would be very useful to have a key midwife and/or obstetrician involved in DART and to liaise with the DART team. It is expected that women will return to the DART clinics around 2 weeks after birth, the Outcome of Pregnancy form should be completed at this time.

Advice to women in DART

Pregnancy is an exclusion criteria for enrolment into DART, also women are advised not to become pregnant during the trial because of unknown long-term effects of ART on infant outcome. However, we must accept that pregnancies will occur and women must be reassured that if they should become pregnant they will continue to receive the medication and clinical support provided by DART. They should feel comfortable to inform clinic staff should they think they may be pregnant and should feel under no pressure to hide or terminate the pregnancy as a consequence of being in DART. If a woman does become pregnant in DART, she then requires advice and counselling about:

1) Continuation of pregnancy
2) Risk of mother-to-child transmission of HIV infection while in DART
3) Management of STI during pregnancy and breastfeeding, if she is randomised to STI
4) ART regimens during pregnancy
5) Blood tests during pregnancy
6) Management during delivery
7) Breastfeeding
8) Follow-up of her baby

<u>Information for the Woman</u>

The pregnant woman should be given written information about the risks and benefits of pregnancy continuation. This should be accompanied by counselling and oral information. Sites will develop and share patient information sheets based on the outlines below.

Most women will have conceived while on ART. They should be counselled about the risk of mother-to-child transmission (which will be low [<2%] if they are well and adherent to medications). They should also be told about possible risks of exposure to drugs to the baby. Explain that although many women in the west have had babies while taking ART with no problems, we do not know a lot about the effect of drugs on babies, especially in the long term. The information sheet should also contain written information about any proposed changes to ART regimens in pregnancy including management of STI, proposed follow-up and management during pregnancy and at delivery, management and any changes to her of ART regimen as a result of pregnancy, follow-up after delivery (including breast-feeding) and follow-up of the baby.

Pregnancy reporting in DART

Any woman who becomes pregnant in DART should have this reported using an SAE form (note that pregnancy is not considered an SAE, just reported on Form 10) with the event given as pregnancy (code 60.01) and indicated as type: pregnancy. At the end of pregnancy (whether live birth, still birth or abortion) <u>a "resolution" SAE form does **not** need to be</u>

completed. Instead, at the end of a pregnancy (whatever the outcome), an Outcome of Pregnancy form (Form 29) should be completed.

It may be that some abortions/deliveries count as SAEs for the mother according to the criteria in section 8.8.4. In such cases an additional (initial) SAE form should be completed at the time for the adverse event that is serious (rather than the pregnancy itself), with further follow-up or resolution forms as appropriate.

Outcome of Pregnancy form (form 29)
The Outcome of Pregnancy form asks some questions about storage of specimens from the baby. We do not require specific samples to be taken from babies but would like to know if samples have been stored as part of clinical care. Similarly HIV-testing of babies is neither provided nor requested by DART but if testing has been carried out and the mother is willing to share the result we would like to know the result.

Time in labour should be recorded as the approximate number of hours from the onset of labour to delivery.

Antiretroviral drugs in pregnancy

If the woman decides to continue with the pregnancy, her ART medication should be reviewed as follows:

First-line regimens:

ZDV+3TC+NVP	There is a lot of experience with these drugs in pregnancy, no need to change from this regimen.
ZDV+3TC+ABC	There is limited experience of Abacavir in pregnancy, as it has generally been used in women with limited other options. There are no clear data to support a change from ABC if the mother is well established on the regimen and has had no side effects. GSK feel unable to recommend use of ABC in pregnancy due to lack of data. In individual cases ABC could be swapped to NVP if a clinician had particular concerns. Note: ABC is being continued in quite a number of pregnancies reported to the UK register.
ZDV+3TC+TDF	There is limited experience of TDF in pregnancy. In Europe, it is mainly used only in women with limited other options. There have been some concerns raised from animal experiments about bone effects. No specific human foetal effects have been reported so far. There are no infant PK data. Women could be given the option to substitute NVP for TDF. TDF is generally being continued in pregnancies reported to the UK register.
D4T	If a woman is taking d4T (i.e. ZDV was replaced for toxicity), consideration could be given to replacing this again with ZDV, mainly because the latter has proven efficacy for prevention of MTCT. However, the risk of anaemia and risk of bleeding associated with the proposed place of birth must also be taken into account before this decision is made. Thus this decision should be made on an individual basis.

Second-line regimens

ddI+d4T	is contraindicated in pregnancy because of the risk of lactic acidosis and should be appropriately replaced by another NRTI combination (the easiest is likely to switch back to ZDV from d4T)
ddI	OK if not with D4T

EFV	should be switched to NVP (in fact there is an argument for using NVP instead of EFV if there is a risk of pregnancy).
KAL	is being used in Europe but there is less experience than with some other PIs.

Women should be encouraged to adhere well to their ART regimen and to continue to take all their medication, and not to miss any doses.

Pregnancy and DART randomisations

CMO/LCM

Whether the woman is in the CMO or LCM arm, pregnancy would normally be a clinical indication to undertake a full blood count and haemoglobin as per normal ANC practice. If a woman is receiving NVP a clinician may consider requesting LFTs for a woman in the CMO arm.

In addition, routine tests such as VDRL, STD screening, urine testing etc. should be preformed according to local antenatal practice. Tetanus toxoid and iron/folate should also be administered as per national ANC guidelines. These tests will normally be provided by the woman's ANC clinic rather than the DART clinic.

For women in LCM, it should be noted that CD4 counts fall during pregnancy because of plasma expansion. This should be born in mind when considering the possibility of regimen failure.

STIs

Pregnancy and breastfeeding are exclusion criteria for the STI randomisation.

As shown on the flow sheets a pregnancy test should be carried out for all women before the start of any STI. If the result is positive they should not commence the scheduled STI.

Should a woman be found to be pregnant during an STI this will normally be during the first trimester. Such women should be told that they should go back onto ART and take it continuously during pregnancy and breast-feeding. Restart of ART may be delayed until after the first trimester, if the woman is well. STIs should only be recommenced after breast-feeding has ceased.

Ante-natal care (ANC) / management in pregnancy

Ante-natal care and infant feeding counselling should be provided as per national guidelines (reference documents from respective ministries of health).

PMTCT information will need to be modified in view of the ongoing ART to the mother and the ART drugs (dosages and duration) we intend to use in the newborn infant.

Women will normally be referred to an antenatal clinic. However, it may be useful, if there are reasonable numbers of pregnant women, for them to be reviewed in the DART clinic with a midwife or obstetrician and receive ANC in DART. The DART clinic should ensure that the woman informs their antenatal clinic that they are enrolled in DART. Sites may chose to provide a referral letter for the woman to give to their ANC clinic giving some details of DART including the ART regimen. There should also be details of how the clinic can contact the DART team for further information. Sites may choose to develop links with ANC clinics to facilitate patient management and exchange of information.

The frequency of ANC visits will depend on the stage of the pregnancy and should follow local practice. DART visits should continue as per normal during pregnancy, and follow-up nurse and doctor forms should be completed in the normal way.

Malaria in pregnancy
Malaria infection in pregnancy poses a substantial risk to the mother, the foetus and the newborn infant. Pregnant women are less capable of coping with and clearing malaria infections. In areas of low transmission of P. falciparum, where levels of acquired immunity are low, women are susceptible to attacks of severe malaria, which may result in stillbirths or spontaneous abortions, or the death of the mother. In areas of high P. falciparum transmission, levels of acquired immunity tend to be high and women may have asymptomatic infections, which may result in maternal anaemia and placental parasitaemia. Both of these conditions can lead to low birth weight, an important contributor to neonatal mortality.

Sites in Uganda (where malaria is endemic) and in Zimbabwe if women are travelling outside Harare should consider implementing intermittent treatment with 3-dose treatment regimens of sulfadoxine-pyrimethamine: once in the second and twice in the third trimester to prevent malaria in pregnancy. In HIV-positive women, the 3-dose treatment is significantly more efficacious than the 2-dose regimen. Such prophylaxis may be provided by the antenatal clinics.

Management of HIV infection during pregnancy
The normal DART guidelines with respect to clinical and/or CD4 should be followed for switching ART. However, as noted above, ***CD4 counts normally fall during pregnancy***, so this should be borne in mind when considering switching for immunological failure in LCM patients. Note also that early side-effects of a new ART regimen may be less tolerable in pregnancy.

Management at Delivery
IT IS VERY IMPORTANT that the pregnant woman and DART team link up with an appropriate (recommended) delivery institution, so that:

1) Midwives/doctor etc are aware that the mother is on ART and the need to continue with it during the peri-partum and the post-partum period.
2) Delivery is actively managed, with avoidance of PROM and foetal scalp electrodes, but with treatment of infections, etc.
3) Midwife or a doctor who supervises the delivery need to be informed about the mother's ART and the infant ART prophylaxis
4) Infant prophylaxis be given at the appropriate time
5) DART team (2 members identified to care for pregnant women (most likely a nurse and a doctor)) to be the key liaison personnel with the place/institution where the mother delivers, to ensure that pregnancy and outcome data are retrieved from the maternity notes as appropriate, blood specimens from the mother and infant are taken and stored if agreed, and to ensure that the mother and infant are given the correct designated ART

It is anticipated that women should attend the DART clinic 10 days to 2 weeks after giving birth. The outcome of pregnancy form should be completed at this time.

Postpartum
The mother should be followed-up and treated according to the DART protocol and MOP. Family planning and counselling services should be offered to all women in the postpartum period as per national guidelines.

Currently infants cannot be followed up under the DART protocol, however, we plan to develop a substudy for follow-up. Infants should be managed (including monitoring for toxicity and infection status) according to local guidelines. Mothers should be counselled about options for HIV testing of infants. Cotrimoxazole prophylaxis should be started at 4 to

6 weeks, regardless of knowledge of HIV status. Although HIV testing of infants cannot be carried out as part of DART the optimal way is to do HIV – DNA PCR, or a viral load test. Minimum testing would be to perform this in any child for whom one had clinical concerns and were considering starting ART before 18 months of age. If available, testing should be done at 6 weeks and again around 3 months after stopping breast-feeding, or according to WHO/national guidelines. Cotrimoxazole prophylaxis should be continued until after breast-feeding has stopped and a negative test-result obtained for the baby.

<u>Breast Feeding</u>
We do not have enough data about breast-feeding while on ART. We hope that breast-milk viral load will be suppressed as the mother is on triple ART, so the risk of transmission would be small. We hope that the level of ongoing ART exposure to the infant would also be small (although this should help prevent transmission during breast-feeding), but this needs to be confirmed with PK studies. It would be very useful to monitor VL and drug levels in breast-milk. However, this needs to be a separate substudy (part of PK substudy or could be part of follow-up of infant protocol) and would need consent, so cannot be implemented at present.

As per national guidelines women should be counselled about the possible risks (exposure of the infant to drugs and possibly to HIV) and known benefits of breast-feeding. The option of rapid weaning to reduce the duration of breast-feeding should be discussed. The transmission risks for women on triple-ART may be considerably reduced compared to women on no ART.

Antiretroviral therapy during delivery and postnatally
<u>Mother</u>
The woman should continue ART throughout delivery as regularly as possible and also in the postnatal period. As the woman is on triple therapy, additional NVP or ZDV for prevention of MTCT (as per local guidelines) should NOT need to be given to the mother.

<u>Infant</u>
The most appropriate prophylaxis for the baby born to a mother taking triple ART is not completely clear and there are no trials to inform this decision. In Europe and US, babies are generally given one or more of the same drugs that the woman is receiving, and in particular, receive ART known to reduce MTCT (e.g. NVP if the mother is on NVP or ZDV +/- 3TC if the mother is on these NRTIs). However, this is in a setting where most women have VL carefully monitored and will have VL below the level of detection. In the DART trial the main danger for transmission is for women who have detectable virus and are failing their regimen.

Therefore there is an argument that prophylaxis for babies is best undertaken with a different drug to those the woman is receiving. ***For women on first-line TDF+CBV, NVP should be given to THE INFANT ONLY WITHIN 4 HOURS OF BIRTH, as it will not have been given to the mother.*** Babies could also receive ZDV+/-3TC for 1 week as well as this single dose of NVP, ***starting within 4 hours of birth.*** However, giving all 3 may be difficult as liquid formulations have to be obtained. NVP could be given as part of a national program.

For mothers taking NVP+ZDV+3TC, NVP post-exposure prophylaxis should be given for the baby starting at 48 hours. ZDV+/- 3TC could be added for 1 week, if this is feasible and available, as there are few other alternative regimens.

Sites may deviate from this policy if individual circumstances indicate an alternative, but should inform the MRC CTU of their intentions.

Patient information sheets
Here are some ideas, please annotate / change to more appropriate language etc.

Pregnancy in the DART trial

As a woman in the DART trial you will have been advised against pregnancy because of the slight possibility that your anti-HIV drugs might harm your baby. However, for most of these drugs there is no clear evidence that they may cause any problems. If you are taking a drug that is known to have a risk your doctor will change that to another anti-HIV drug. If you have become pregnant you will continue to receive anti-HIV drugs within the DART trial as well as continued clinical care and support.

<u>What is the risk of my baby being infected with the HIV?</u>

If a mother is not receiving any treatment and she breastfeeds her baby then there is at least a one in three chance the baby will be infected with HIV. The risk to the baby can be higher if the mother is sick with a high level of virus in the blood.

If a mother is receiving anti-HIV treatment, as you are in the DART trial, and not missing any doses, then there is likely to be a very low risk of the baby being infected with HIV (only around one in one hundred in women who do not breast-feed, and may also be as low in women who continue on treatment whilst they breast feed, but we do not know for sure yet).

<u>Will taking anti-HIV treatment in pregnancy cause any harm to my baby?</u>

Until now there have been no reported increases in any kind of birth abnormalities in babies born to women taking ART in pregnancy. However this is still something that is being closely looked at. We know that some of the drugs may cause problems to babies if the mother is taking them during pregnancy and your doctor will discuss changing your drugs if you are taking one of these medicines:

Some drugs have been used a lot in pregnant women and we think they are probably safe, although the long-term effects are not yet known.

Combivir+Nevirapine: Many women have become pregnant around the world whilst taking this combination: Most have remained very well, but some women and babies do get anaemic with ZDV.

Combivir+Abacavir: There is less experience of abacavir use in pregnancy, so far no specific problems have been reported. Your doctor may recommend switching from ABC to NVP.

Combivir+Tenofovir: There is less experience of TDF in pregnancy. So far no specific problems have been reported in babies. Concern that TDF could affect the baby's bones has been expressed because it has been seen in some baby animals whose mothers received TDF.

Other drugs:

Efavirenz: This is known to cause abnormalities of the brain in animals, although not reported in humans. If you are taking this drug, your doctor will want to change it as soon as possible.

Stavudine + These should not be given together to pregnant women as it may

didanosine: make them sick, especially late in pregnancy. Your doctor will probably change one of these drugs if you are taking them.

Kaletra: If you are taking Kaletra your doctor will recommend that you continue to take it throughout pregnancy.

Long-term follow-up of babies born in DART
As there may be rare effects of the treatment that we do not yet know about, you may be asked to join a long-term study so that we can follow up your child and make sure that they remain well over the next few years.

What do I do about my ART when it comes to the delivery of the baby?

Its very important to continue to take your medicines throughout pregnancy and not miss any of your doses, this is the best way to keep you well and to have the smallest risk of HIV passing to your baby. This is especially important around the time of delivery as this is when there is the highest risk of the virus passing to the baby. Always keep your medicines with you and don't stop taking them at any time. Continue to take them at the regular time during labour, and after the baby is born. This is when they are most needed to protect the baby from the virus.

Will my baby need to take any medicines?

Your doctor may recommend the anti-HIV drugs for your baby shortly after birth. This may include NVP being given to your baby (but not to you) as part of the national program. Your doctor will tell you what your baby will need to take and when.

You should also be offered cotrimoxazole for your baby from 4 to 6 weeks after birth. If your baby should become HIV infected despite the low risk, this drug would help protect your baby from other infections which are common in babies with HIV infection.

Is it safe for my baby if I continue on my ART whilst I breast feed?

It is likely that the risk of HIV infection passing to your baby whilst you breast-feed whilst taking ART will be very small. However, we do not know this for sure as studies to look at this more closely are only currently underway. We may ask to collect samples of breast-milk to check for levels of the anti-HIV medicines in breast-milk, as very little is known about this.

You will receive advice from doctors and nurses about HIV transmission and breastfeeding. If you breastfeed, you will be given advise about when to stop and about mixed feeding. [sites to add/modify as appropriate]

Much more information about the safety of breast-feeding on ART will come out over the next few years, ask your nurse / doctor for an update.

How do I find out whether or not my baby is infected with HIV?

[sites to add/modify as appropriate]

Can my baby receive treatment if he/she is infected?

[sites to add/modify as appropriate]

Other questions

You may have other questions about being pregnant on ART? Please don't hesitate to ask your nurse / doctor who may be able to answer them. They may also be able to put you in touch with another woman who has been pregnant / had a baby on ART so you can talk about the experience with her.
